# Autoimmune pemphigus: difficulties in diagnosis and the molecular mechanisms underlying the disease

**DOI:** 10.3389/fimmu.2025.1481093

**Published:** 2025-03-03

**Authors:** Olga Simionescu, Sorin Ioan Tudorache

**Affiliations:** ^1^ 1^st^Clinic of Dermatology, Carol Davila University of Medicine and Pharmacy, Colentina Hospital, Bucharest, Romania; ^2^ Department of Preclinical Disciplines, Faculty of Medicine, Titu Maiorescu University, Bucharest, Romania

**Keywords:** pemphigus, direct immunofluorescence, bullous diseases, desmogleins, diagnostic

## Abstract

Recently recognised as a desmosomal disorder, autoimmune pemphigus remains severe in some of its forms, such as pemphigus vulgaris. This review is divided into four parts. “Cellular and molecular mechanisms in autoimmune pemphigus” discusses in detail antigenic targets, antibodies, immunological and genetic mechanisms of apoptosis and the involvement of cells and organelles (keratinocytes, lymphocytes, eosinophils and neutrophils) in different forms of pemphigus. These advances have led to today’s first-line biologic therapy for pemphigus. The section “Specific features in the diagnosis of immune pemphigus” deals with the clinical diagnostic clues (enanthema, intertrigo, pruritus, distribution of lesions). The third section, “Characteristics and challenges in different types of pemphigus”, focuses on the importance of using standardised diagnostic criteria in paraneoplastic pemphigus and pemphigus herpetiformis, the specific and difficult situations of differentiation between bullous lupus and autoimmune Senear-Usher pemphigus, between IgA forms of pemphigus or differentiation with other autoimmune diseases or neutrophilic dermatoses. The possibility of subtype cross-reactivity in pemphigus is also discussed, as is the diagnosis and course of the disease in pregnant women. The final section is an update of the “gold standard for the diagnosis and evaluation of autoimmune pemphigus”, the role and place of direct immunofluorescence and additional serological tests. This revision is the first to combine the difficulties in clinical diagnosis with new molecular insights. It provides a comprehensive overview of recent advances in the understanding of autoimmune pemphigus, bridging the clinical challenges and complexities of diagnosing different forms of pemphigus, and is a valuable resource for clinicians caring for patients with pemphigus.

## Introduction

Autoimmune pemphigus is a group of acquired autoimmune bullous diseases of the skin desmosomes (1, 2). Some clinical forms, such as pemphigus vulgaris (PV), can be severe and have a guarded prognosis. Classically subdivided into deep pemphigus (*vulgaris* and *vegetans*) and superficial pemphigus (*foliaceus*) according to the epidermal site of acantholysis, recent years have brought precision in the characterization of forms of autoimmune herpetiform, IgA, paraneoplastic pemphigus. The recognition of clinical manifestations in a field of considerable difficulty for the clinician (wherever he is in practice and regardless of whether he is a general dermatologist or from a tertiary autoimmune disease service) is essential for correct classification and effective treatment.

New immunologic therapies, such as checkpoint inhibitors, target different I mmunologic mechanisms than those originally described in post-drug pemphigus (3). The immune form of pemphigus is distinguished from the benign familial Hailey-Hailey form by mechanism of production, genetic transmission, and prognostic criteria (4). As an important public health problem, immune pemphigus has been reported in all ethnic groups, but susceptibility in different populations correlates with positivity for certain Human leukocyte antigens (HLAs) with respect to geographic areas or population categories. Major laboratory advances in diagnostic authentication have been driven by advances in the understanding of immunological mechanisms. Research is needed today to see what is the possibility of crossover between different forms of immune pemphigus and genetic predisposition, with consequences on the prognosis and impact on therapeutic protocols.

This paper is a review with a double perspective, clinical and immunologic. Its aim is to summarize the most difficult challenges in the understanding of the mechanism of the disease and to represent a valuable tool for the practicing dermatologist or internist when confronted with difficult cases of pemphigus, since it is the first approach to the diagnosis of the disease.

## Cellular and Molecular mechanisms in autoimmune pemphigus

**Figure 1:**
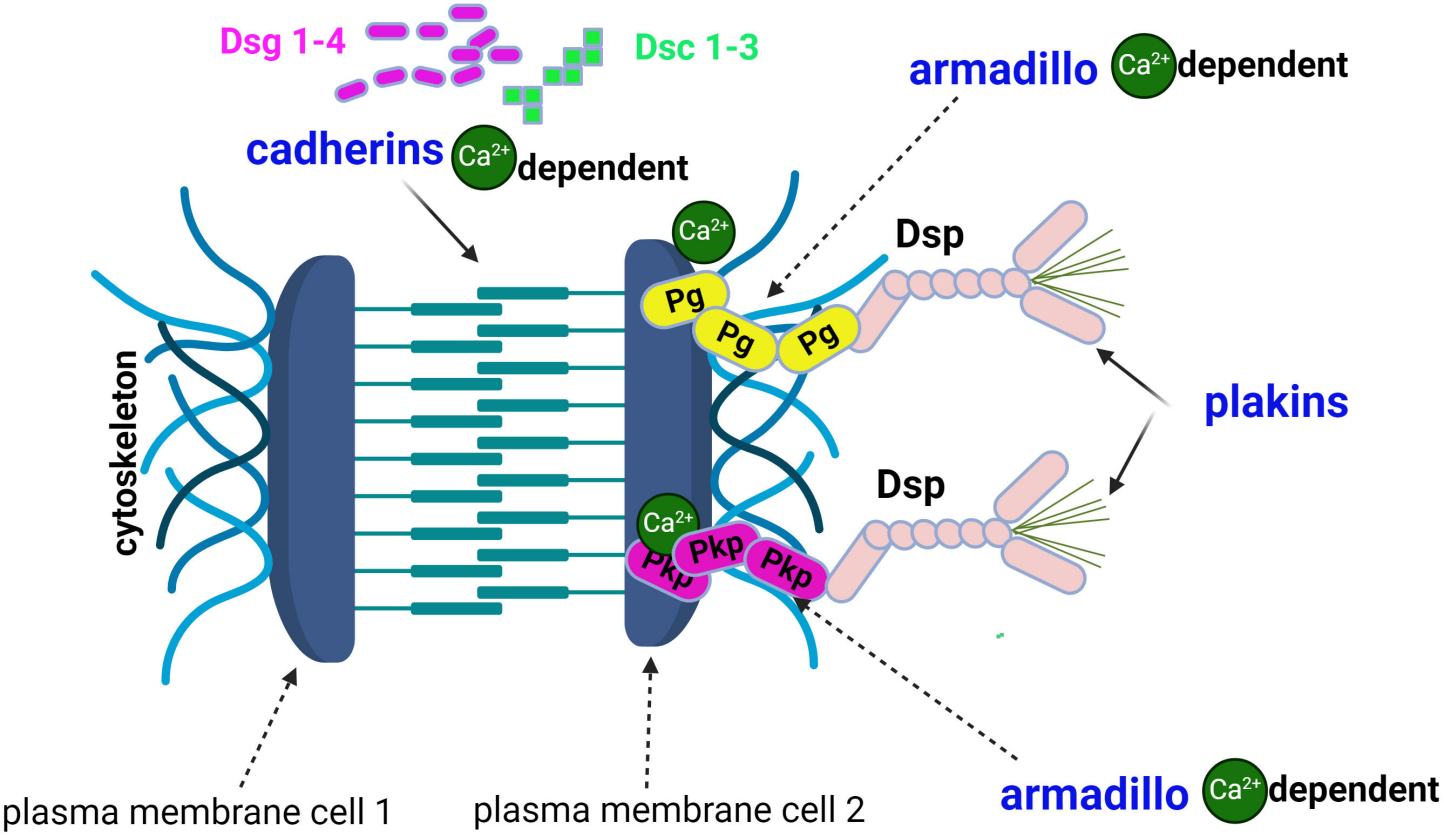
The structure of the desmosome comprises 3 families of proteins: cadherins, *armadillo* proteins and plakins. Desmogleins (Dsg1-4) and desmocolins (Dsc1-3) are calcium-dependent cadherins. The *armadillo* protein family includes plakoglobin (Pg) and plakophilins (Pkp), which are also calcium-dependent. Desmoplakin (Dsp) and plectin are plakins and link cytoskeletal structures.

*Desmosomes* are the key element of cell-cell adhesion complexes (5) and are composed of three protein families (**Figure 1**): cadherins (6, 7), *armadillo (* 7) proteins, and plakins. The first two families are calcium dependent (7, 8). The cadherin superfamily includes the *desmogleins* (Dsg1-4), the *desmocollins* (Dsc1-3) (7, 9), and exhibits a unique dependence on extracellular Ca^2+^ to rigidify the extracellular domains and allow homophilic interactions (6). Through their extracellular N-domain, desmosomal cadherins form -*cis* and -*trans* interactions (7, 8). with their homologs on the same or neighbouring cells to form knot-like structures with desmosomes. The *-cis* interactions are the weakest. The -*trans* dimers required for cadherin activation are dependent on Ca^2+^ions, which also exert a protective anti-proteolytic effect. Cadherins (**Figure 1**) are involved in cell polarisation and proliferation *via* osmotic pressure within the keratinocyte and exert functions in cell adhesion (5, 8, 10) by reducing tension and establishing the cell-cell contact. Cadherins also stabilise this contact by resisting the physical forces that pull on the contact (5, 10). There are two main hypotheses (11) regarding the loss of cell-cell adhesion: *the steric hindrance theory* and *the signalling theory* (5, 7, 11). The former is based on the direct interference or transinteraction of IgG with Dsg3 and the latter on the activation of signalling pathways: cellular-multifunctional transcription factor (c-Myc), p38 mitogen-activated protein kinases (p38MAPK), Ras homologue family member A (RhoA).

The *armadillo* protein family (7) includes plakoglobins (Pg) and plakophilins (Pkp) (**Figure 1**).

The third family of desmosomal proteins are the plakins, including desmoplakin (Dsp) and plectin (7) which link cytoskeletal structures together.

In addition to their essential role in keratinocyte adhesion, desmosomes are also signalling hubs (10). An equivalent is found in the heart, in cardiomyocytes (2), where intercalated discs are composed of desmosomes and *adherens* junctions. In desmosomal heart disease the result is an arrhythmogenic cardiomyopathy (2), whereas in the skin, the damage to keratinocyte cohesion leads to the formation of vesicles/bullae by *acantholysis* (acantha, *gr.* spine, lysis, *gr*. rupture), a process underlying the development of pemphigus. Incidentally, the same mixed desmosome-*adherens* junction is initially present in the epithelium as in the umbilical cord. However, it is only transiently present in the formation of demosomes, which are ultimately responsible for the tight cohesion of the keratinocytes (12) to maintain the integrity of the barrier function.

**Figure 2:**
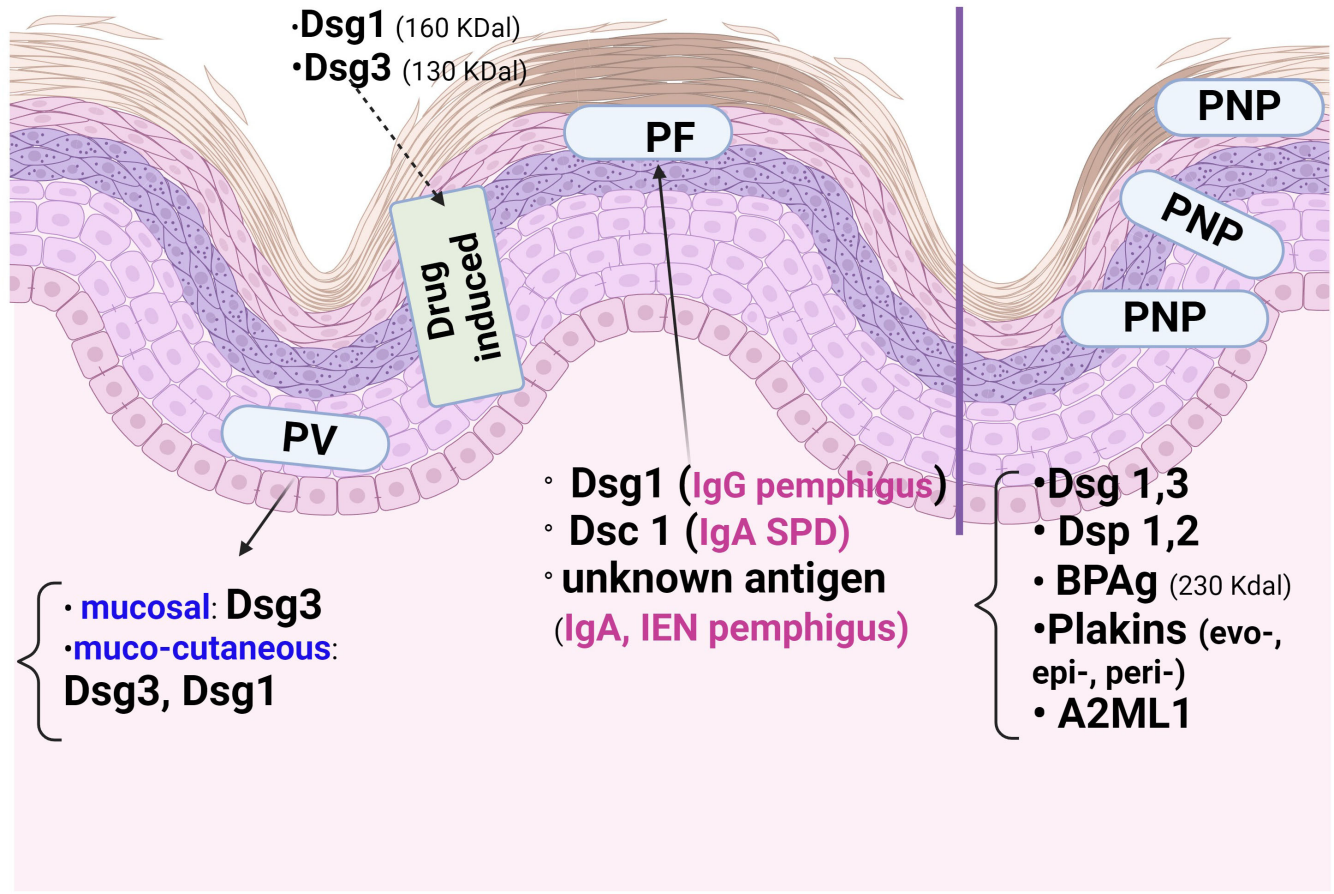
The epidermal distribution of pemphigus antigens varies according to the type of autoimmune pemphigus and the site of acantholysis. The mucosal form of Pemphigus Vulgaris (PV) has Desmoglein3 (Dsg3), whereas the cutaneo-mucous form has Dsg3and Dsg1. Paraneoplastic Pemphigus (PNP) has many antigens, including those of Pemphigoid bullous (PB), which explains the Direct Immunofluorescence (DIF) picture. In Pemphigus foliaceous (PF), Dsg1 and Dsc1 predominate. In the immune form of drug pemphigus, Dsg1 and Dsg3 are the proteins involved in acantholysis in thiol-containing drugs.

**Figure 3:**
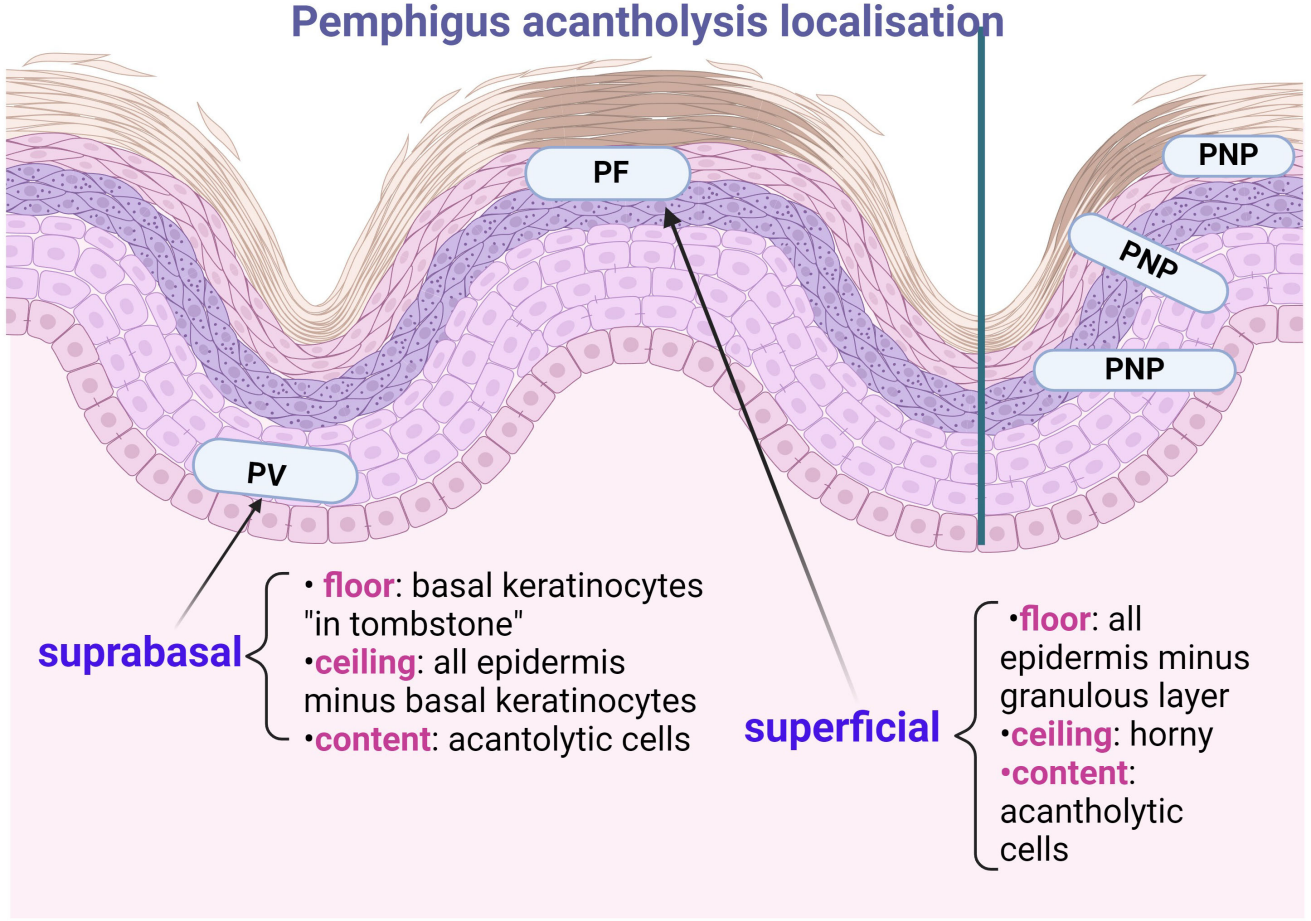
Importance of localising acantholysis in pemphigus: Pemphigus Vulgaris (PV) shows suprabasal acantholysis, with basal keratinocytes in “thumb stones”, the prognosis of patients before the era of corticosteroid therapy being that “of histopathological examination”. Pemphigus foliaceous (PF) has superficial acantholysis in the stratum corneum or subcorneum and has a better prognosis. Paraneoplastic Pemphigus (PNP) may show interface dermatitis (one of the first Anhalt criteria) or acantholysis at any epidermal level. The ‘ceiling’ and ‘floor’ of the acantholytic lesion should be specified, the contents being the fluid in which the acantholytic cells float.

Dsg1, Dsg3, Dsc1-3, mitochondrial proteins (1, 9) and subtypes of the acetylcholine receptor are the major ***antigens*** of pemphigus (**Figure 2**). The most commonly targeted antigens are the Dsg1 (160 KDal) and Dsg3 (130 KDal) proteins, which have two domains: extracellular and cytoplasmic (5, 6). In autoimmune pemphigus, acantholysis is an immunological process that occurs as a result of structural damage to the desmosome, and the site of acantholysis in the epidermis correlates with the severity of the disease form (**Figure 3**). For example, suprabasal acantholysis is characteristic of severe disease (PV), while superficial acantholysis (granular epidermal layer) is of interest in moderately progressive forms, such as pemphigus foliaceus (PF).

The clinical phenotypes of pemphigus depend on the autoantibody profile (13, 14) and the target antigens, which are mainly Dsg1 and/or 3 in PV and Dsg1 in PF. Dsg1 and Dsc3 are differentially distributed in the epidermis (**Figure 2**), with Dsg1 and Dsc1 more prominent in the superficial epidermal layers, whereas Dsg3 and Dsc3 dominate the deeper layers (11, 13). Therefore, in contrast to the mucosal dominant form of PV (13), which has antibodies to Dsg3 only and is restricted to the mucosa, the mucocutaneous form of PV has antibodies to Dsg1 and Dsg3 and affects the epidermis and mucosa. At the corneous layer, both Dsg1 and Dsg3 are restricted to desmosomes (corneodesmosomes) (13), whereas Dsg co-localisation with Pg differs extradesmosomally: Dsg3 co-localises with Pg, whereas Dsg1 has the same extradesmosomal co-localisation with Pg in all epidermal layers.

**Figure 4:**
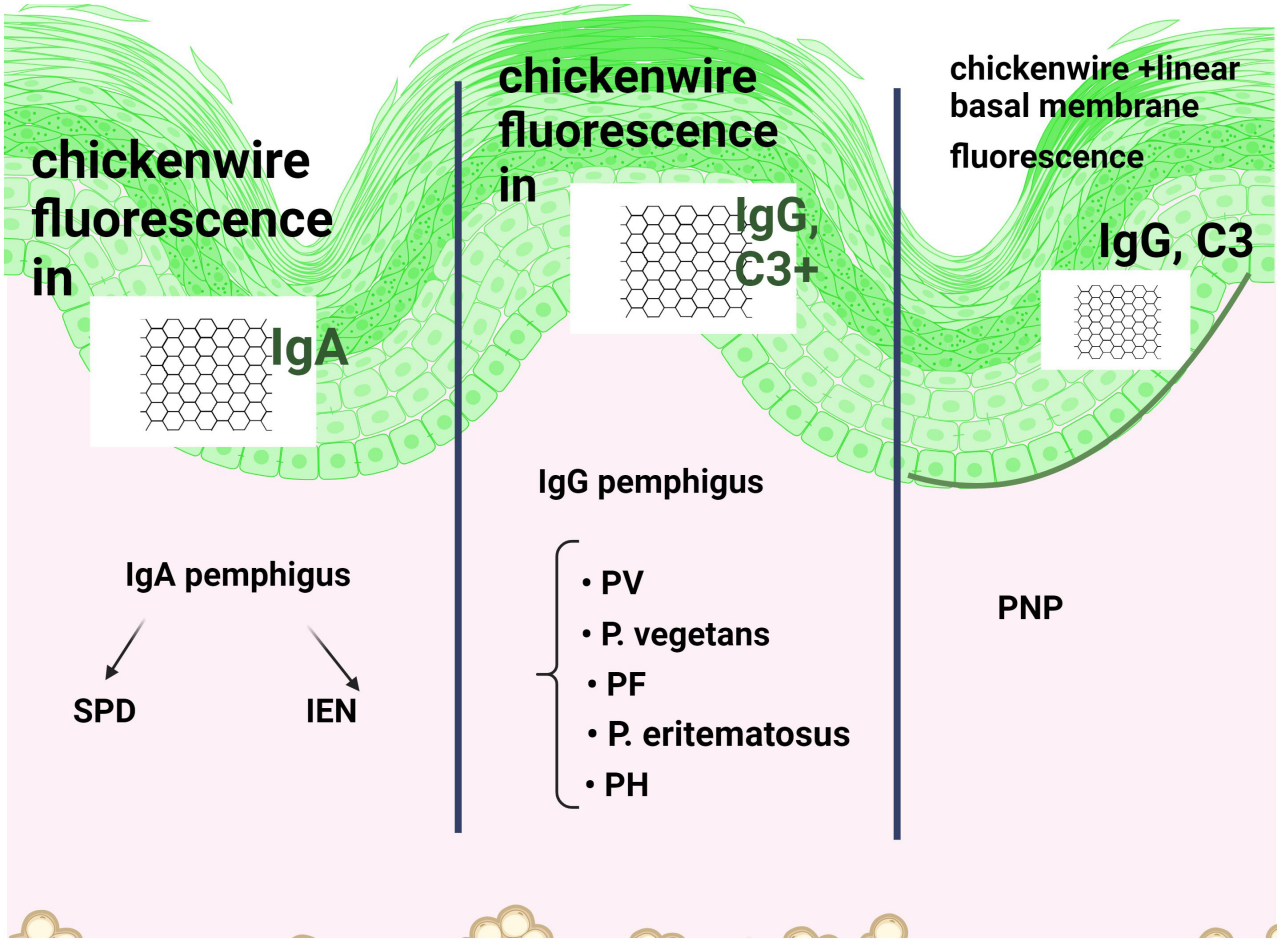
Direct immunofluorescence (DIF) in immune pemphigus shows reticular intraepidermal fluorescence when IgG or IgA antibodies or C3 are positive on the cell surface of keratinocytes. While FITC is a common green fluorescent dye, other conjugates are also used. The site of acantholysis is not noted, only the fluorescent aspect. IgG and IgA pemphigus are distinguished according to the (fluorescently labelled) antibody that fluoresces positively. Due to the presence of several antibodies, including Pemphigoid bullous (PB), additional linear, continuous basement membrane fluorescence in IgG is associated with Paraneoplastic pemphigus (PNP).

*Autoantibodies* in autoimmune pemphigus are of the Ig G (more common) and IgA types (5, 14) (**Figure 4**). *IgG4* are found in PV and PF and are known as”IgG4 autoimmune diseases (IgG4-AID)” (15), which include *myasthenia gravis*, nodo-paranodopathies with autoantibodies against paranodal and nodal proteins, and encephalitis with antibodies to LGI1/CASPR2.

The pemphigus patient develops IgG autoantibodies by converting IgM to IgG (15, 16), a process that correlates with the pemphigus phenotype and is made possible by the poorly flexible pentameric structure in the Fab region of IgM, which does not allow it to interact strongly with tight junctions. In healthy individuals, the B cells (16, 17) prevents the production of anti-Dsg3 autoantibodies IgG at the inhibitory Fc receptor (FcΔRIIB). Anti-Dsg3 antibodies are necessary to deplete desmosomes to weaken cell adhesion, but they cannot abolish it completely.

The importance of anti-Dsc autoantibodies (9, 12) in pemphigus is based on their essential role in adhesion (5, 10). In the presence of Dsg isoforms 1-4, Dsc-1 becomes the target antigen for IgA autoantibodies in subcorneal pustular dermatosis (SPD). In contrast, autoantibodies directed exclusively against Dsc may be present in various atypical variants of pemphigus.

Although the mechanisms of vesicle induction in pemphigus (13) are not fully understood, the development of the two theories described above (steric hindrance and the signalling) represent important advances (5, 7, 11) in explaining autoantibody production. Ca^2+^-mediated signalling (5) is important for vesiculation and depends on the autoantibody profile, resulting in different roles for the signalling (18) complexes organised by Dsg1 and Dsg3 (6, 14, 19). Pemphigus antigens are triggers for soluble factors of innate (18) immunity such as -*fas* ligand. In more advanced stages, caspases are involved in basal keratinocyte shrinkage and complete desmosome segregation, followed by local apoptosis (1, 20) of the resulting acantholytic cells. Interference with phospholipase C (PLC) γ1 and Ca^2+^signalling (5) may be a promising therapeutic approach.

*Cells involved in immune pemphigus* are keratinocytes (1, 6, 13), B cells (16, 21, 22), T cells, eosinophils and neutrophils (PMNs) (22).

*Keratinocytes* provide a hyperadhesive (6), strong, protective status against IgG attack. Following the process of immune acantholysis, the spiny keratinocyte becomes an acantholytic cell and floats inside the vesicle/bulla. Thus, the acantholytic cells are the same keratinocytes that have lost their cohesion within the epidermis. Keratinocyte apoptosis follows acantholysis and is a limited process recently termed “apoptolysis” (1, 20). One of the 12 cell death pathways described by the Nomenclature Committee on Cell Death (NCCD) (1, 20), the apoptosis of keratinocytes is based on the pathogenic diversity of autoantibodies, mitochondrial dysfunction and p38MAPK signalling.

*B cells* are produced and selected in the bone marrow. How they change as they circulate in the body and the pathway leading to the transformation of a normal immune system into a pathogenic one in PV and FP (17) is being investigated. B cells produce autoantibodies, the levels of which correlate with the number of autoreactive B lymphocytes in the different Dsg fragments (21, 22). Increased levels of B cell activation

*T cells* are characteristic of the predominantly lymphocytic inflammatory infiltrates (22) associated with the dominant mucosal phenotype in pemphigus (22, 23) and their role is pro-inflammatory (Th1) via IFN-γ and Th17. Thus, Th1 mediates this pro-inflammatory response *via* Th2 cell-derived cytokines such as IL-4. For this reason, Veldman considered pemphigus to be a “Th2-dependent disease”, as early of 2006 (24). The loss of balance between regulatory T (Treg) and T helper 17 (Th17) leads to loss of tolerance against desmoglein (Dsg)-3 resulting in pemphigus vulgaris (PV) (25). Recent studies indicate that IL-6, IL-8 and IFN-γ are secreted by CD4+ T cells in co-culture with TNK CD56+CD3- natural killer T cells (NKT cells) (25).

Regulatory T cells suppress the activation of autoreactive CD^4+^ T cells and help to control inflammation, while follicular T helper cells (Th cells) interact with B cells and facilitate the production of autoantibodies, the p38 MAPK pathway and vice versa. The novel subset of CD^4+^ Th follicular CD^4+^ Th cells help to activate B cells, and this interaction between autoreactive T and B cells (**Figure 2**) is essential for humoral autoimmunity against Dsg3. This tandem of T and B lymphocytes lesionally and perilesionally infiltrating the integument of patients with autoimmune pemphigus has been compared to a tertiary lymphoid organ (TLO) originally described in the spleen and lymph nodes (23, 25, 26).

The role of autoantibody/antigen interaction in triggering signalling pathways such as p38MAPK has been questioned but targeted therapy of pemphigus to block this pathway is now being pursued (27). Arguments for the involvement of this mechanism in the pathogenesis of pemphigus lie in the ability of p38MAPK inhibitors to block the activation of caspase-3 proteinases, which are proapoptotic.

Modern therapies (rituximab) are routinely used in PV and are mainly directed against B lymphocytes, but also against T cells (21).

The presence of a *neutrophil* (PMN) infiltrate indicates an intermediate prognosis of the disease (22), and chemotactic activity is exerted, as in the case of eosinophils, by IL-8 secreted by activated keratinocytes. In IgA pemphigus, PMNs infiltrate the integument, which is why the disease is often classified as a neutrophilic dermatosis (28).

The role of the *eosinophil* in autoimmune pemphigus can be analysed by comparison with its involvement in bullous pemphigoid (BP). In both diseases there is an eosinophilic inflammatory infiltrate, but also eosinophilic spongiosis, an important early stage in histopathological examination. While in BP eosinophils are necessary to drive the immune response against the famous 180 Kdal protein (BP2Ag) described by Walter Lever, in immune pemphigus the pathogenic mechanism does not rely on the direct involvement of eosinophils in the development of the vesicle or bullous lesion (29).

**Figure 5:**
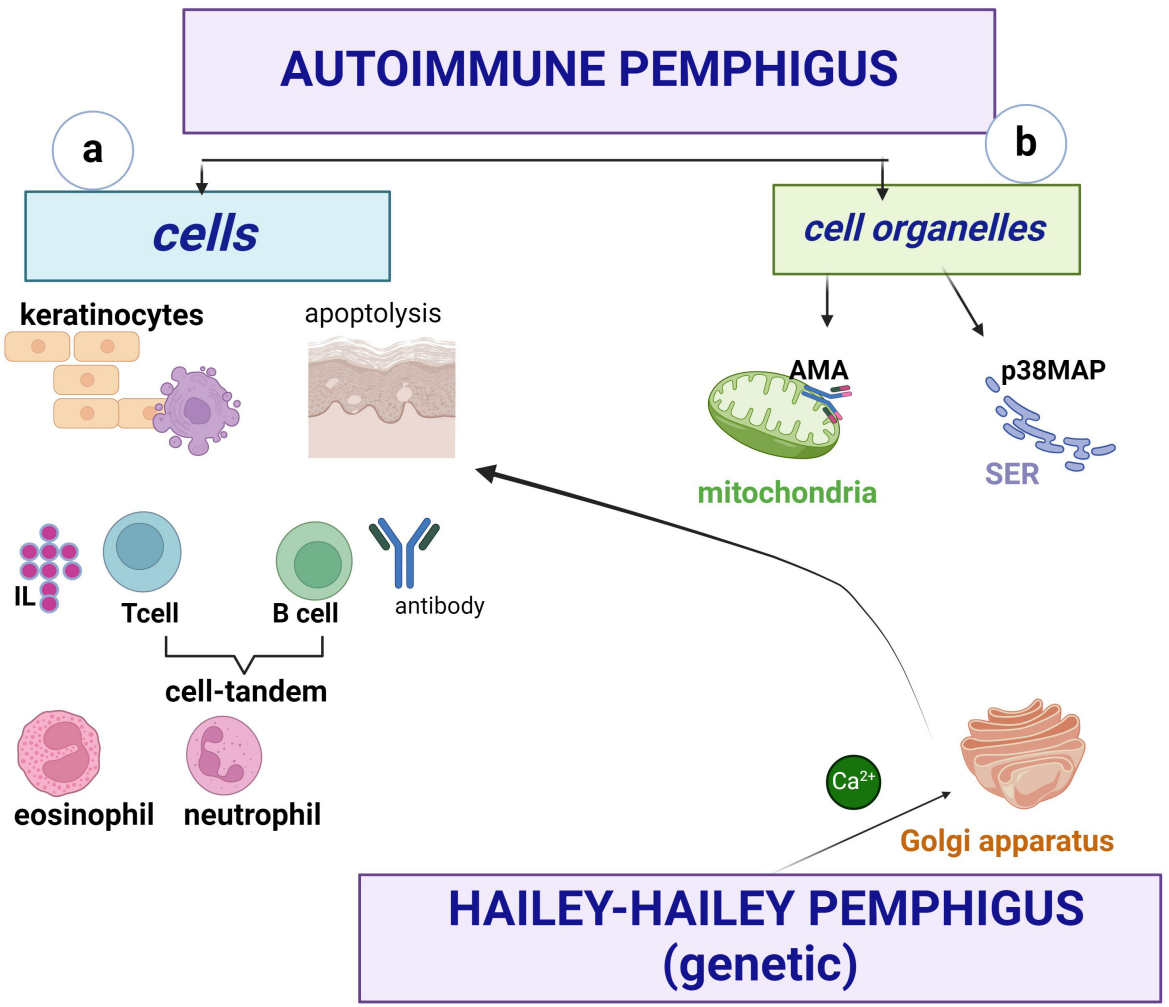
Pemphigus involves cells **(a)** and organelles **(b)**. Keratinocytes apoptoslyze and secrete IL-8, B cells produce autoantibodies and form a tandem with T cells (Th1, Th2, Th17), the latter producing mainly IL-4, IFN-γ. The presence of neutrophils (PMNs) is a prognostic element, and eosinophils are important in the early stage of the disease, called “eosinophilic spongiosis”. At the cytoplasmic level, mitochondria are involved in apoptosis *via* mitochondrial antigens, and at the SER, Ca^2+^ ions are released into the cytoplasm *via* PLC. The Golgi apparatus does not play an important role in autoimmune pemphigus, in contrast to the non-immune form where SPCA1 is a Ca^2+^pump capable of triggering Ca^2+^ influx into the lumen.

*The cellular organelles* involved in *immune pemphigus* are the mitochondria and the smooth endoplasmic reticulum (SER) (**Figure 5**)

Acetylcholine receptors represent *mitochondrial antigens* against which antimitochondrial antibodies (AMA) are developed, causing mitochondrial damage leading to apoptolysis (1, 20). The relationship between AMA and other autoantibodies in autoimmune pemphigus is important, so a good characterisation of their biological behaviour may lead to the development of pharmacological agents to protect mitochondrial function and may serve as a future therapeutic target. Thus, AMA uptake inhibits the ability of PV IgG autoantibodies to induce vesicles/bullae (1, 20), but acts synergistically with other autoantibodies in the pathogenesis of PV.

At the *SER*, Ca^2+^ ions are released from this cell organelle into the cytoplasm *via* PLC (1, 30), hence the anti-acantholytic effect of PLC inhibitors. The role of SER in the activation of mitogen-activated p38 protein kinase in pemphigus signalling pathways is thought to be crosstalk (30) or signalling disruption. The *trans*-compromised interactions of Dsg do not lead to keratinocyte dissociation when p38 MAPK is inhibited (13, 30).

A third cytoplasmic organelle, *the Golgi apparatus*, is not involved in autoimmune pemphigus, in contrast to familial benign non-immune pemphigus (Hailey-Hailey disease), where SPCA1 is a Ca^2+^ pump capable of triggering Ca^2+^ influx into the lumen. The subsequent concentration of peri-Golgi Ca^2+^ signalling is driven to a non-immunological mechanism by mutations in the ATP2C1 gene (4).

## Specific features in diagnosing immune pemphigus

### Importance of enanthema in the diagnosing of immune pemphigus

**Figure 6:**
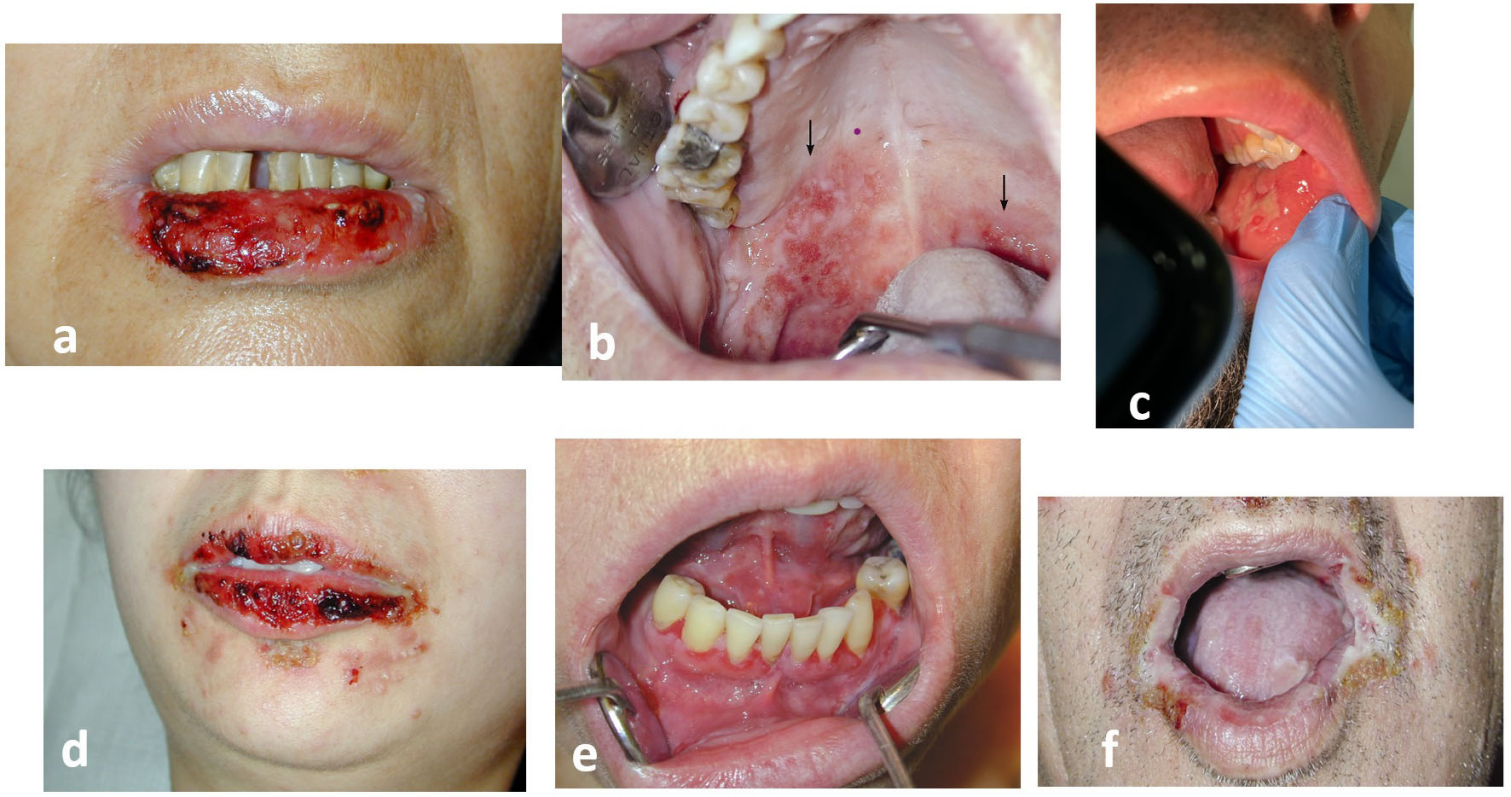
The enanthema of pemphigus: **(a, b)** Pemphigus vulgaris (PV) **(c)**. Pemphigus vegetans (Neumann) **(d–f)**. Paraneoplastic pemphigus (PNP). Always painful, in paraneoplastic pemphigus (PNP) the enanthema has a periorificeal distribution which facilitates diagnosis (authors’ collection cases).

A painful, erythematous erosive enanthema, that interferes with feeding occurs at the onset of PV and precedes the appearance of skin lesions by 6 to 12 months. This painful, often non-specific stomatitis often delays diagnosis, especially when the patient presents to the dentist (**Figure 6**). The delay in diagnosis is 5 months for the erosive-ulcerative form and longer, up to 8 months, for gingival involvement (31). This delay can be attributed to the clinician, the health care system or the patient (31, 32). It is even more important given the high frequency of oral involvement and the easy of oral access (32).

Mucosal lesions prevent feeding and used to be a cause of death. Painful enanthema may also occur in the oesophagus, conjunctiva, nasal mucosa or genital mucosa (vagina, vulva, penis, anal). Spread to the larynx causes hoarseness of speech. The bullous enanthema of immunological pemphigus is not associated with scarring due to its epidermal localisation as a result of achantolysis.

The gold standard for the diagnosis of enanthema, even retrospectively (33), is histopathological examination (**Figure 3**) and direct immunofluorescence (DIF) IgG and/or C3 positivity (33) (**Figure 4**), in addition to other serological tests, such as indirect immunofluorescence (IIF) and enzyme-linked immunosorbent assay (ELISA). In the oral cavity, it is preferable to perform two biopsies, one from the tegument of the lesion and the other from the mucosal tissue (34), because the earlier pemphigus is diagnosed, the more effective the treatment (31, 32).

Pemphigus vegetans is characterised by a “cerebriform tongue”, which has been described since 1981 (35). The typical pattern of *gyri and sulci* over the dorsum of the tongue is a well-known sign of P. vegetans, as in the intertriginous involvement (35, 36). Intractable stomatitis with severe periorificial involvement facilitates the diagnosis of paraneoplastic Pemphigus (PNP) (37), whereas there is no mucosal involvement in PF (38).

The severity of mucosal lesions correlates with the distribution of Dsgl-3, which is present throughout the epidermis in the oral mucosa, in contrast to its cutaneous distribution (suprabasal, in the Malpighian layer) (13, 20, 39, 40). In pemphigus, the mucosal-dominant phenotype is associated with lymphocyte-predominant lesional inflammatory infiltrates of the skin (22). In PNP, we note the correlation of enanthema (**Figure 2**) with the presence of both *cadherins* (Dsgl1, 3) and *non-cadherin* proteins (evoplakin, periplakin, bullous pemphigoid1,2 (BP1, 2)antigens, α-2-macroglobulin-like 1(A2ML1)) (13, 41).

The differential diagnosis of mucosal involvement in pemphigus is often difficult and includes other painful (aphthous ulcers, mucous membrane pemphigoid, herpetic stomatitis, polymorphous erythema of the moderate and severe forms) and painless enanthemas (lichen planus, florid oral papillomatosis, lupus erythematosus, oropharyngeal carcinoma).

### Intertriginous lesions: the diagnostic challenge

**Figure 7:**
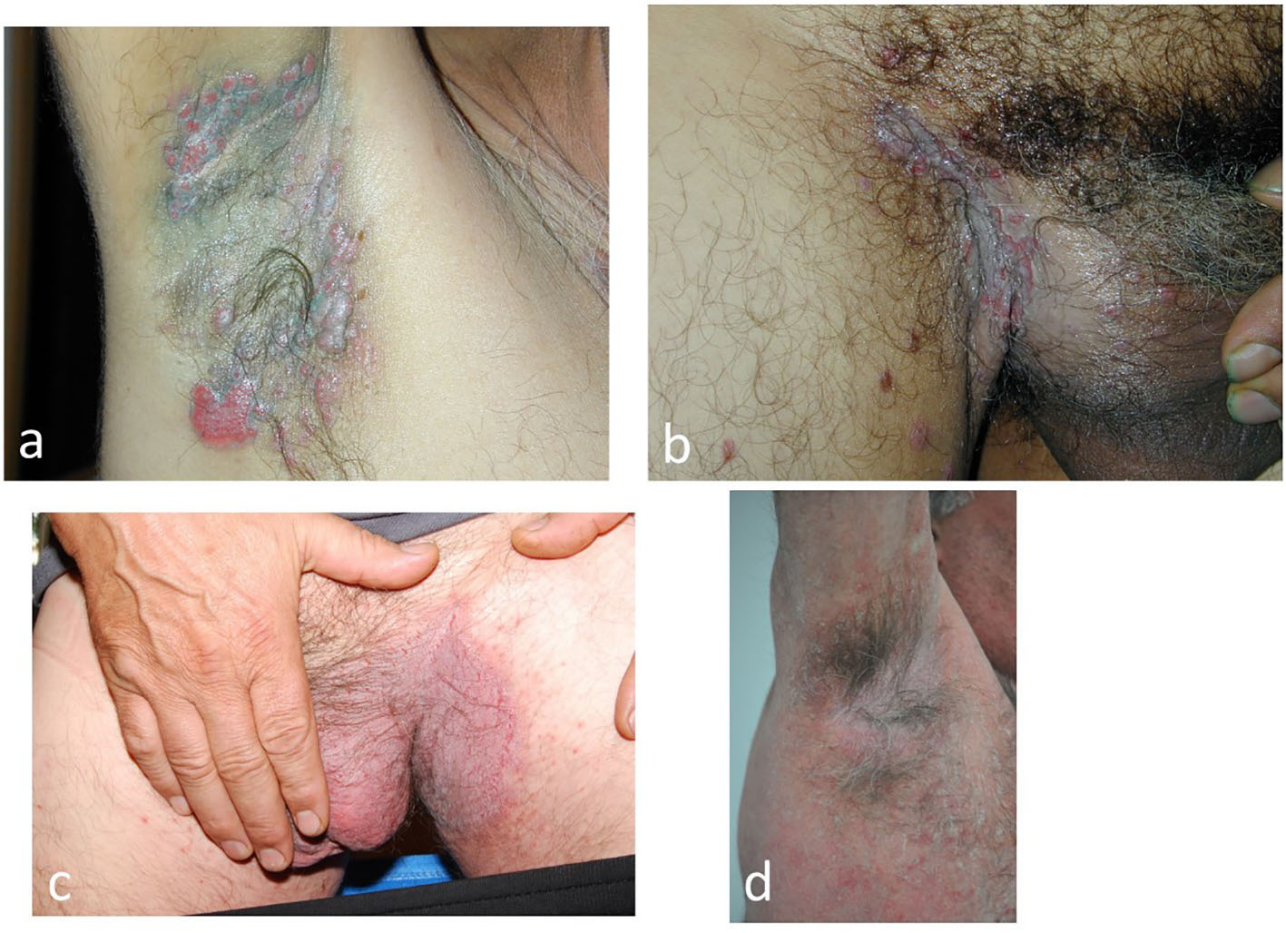
Intertriginous lesions of pemphigus: **(a, b)** Vegetative pemphigus in which the vegetative lesions are initially bullous **(c, d)**. Non-immune Hailey-Hailey pemphigus Hailey (cases from the authors’ collection).

Four types of pemphigus can cause intertriginous lesions: non-immune Hailey-Hailey pemphigus, immune vegetant pemphigus, PNP and the IgA form (**Figure 7**)

*P. vegetans* is initially bullous and rapidly develops vegetative lesions. Patients present with paronychia and nail dystrophy, often with nail haematomas (42). The presence of perivegetative pustules is seen in the moderate form of pemphigus vegetans and helps the clinician to differentiate this variant of Hallopeau’s pemphigus from the severe flaccid bullous Neumann’s disease (43, 44). In *PNP*, intertriginous lesions are not characteristic but may occur. The coexistence of enanthema, with periorificeal distribution and extensive lesions with a targetoid aspect, such as erythema multiforme, facilitates the clinical diagnosis (44, 45). *IgA pemphigus* presents with pruritic vesiculo-pustular lesions in the axillae, groin and proximal parts of the extremities (46).

Personal and family history is valuable in non-immunological Hailey-Hailey pemphigus, an autosomal dominant disease. Accurate diagnosis of intertriginous involvement is achieved by routine skin biopsy and DIF, supplemented by serological testing.

### Pruritus as a symptom of pemphigus

A classical clinical approach divides bullous diseases into pruritic, when there is a “subepidermal bulla”, and non-pruritic, when the bulla is located intra-epidermally. The term “subepidermal bulla” is inappropriate because the location of the vesicles/bullae is at the dermo-epidermal junction, part of the basement membrane.

Three forms of pemphigus may be associated with pruritus, sometimes of increased intensity: *PF* (61%) (47, 48), *PH* and *IgA pemphigus*. The parameter pruritus is related to the severity of the disease (48, 49) and indicates whether the disease is under control or not.

Research is needed in order to elucidate the role of T helper type 2-mediated pathways in comparing pruritus in pemphigus and bullous pemphigoid, BP (49). Other reports have shown a contribution of IL-31 (produced by the activated T-cell) and IL-31receptor α in PF and PH (49). IL-31 is a major pruritogen that has been widely described in atopic dermatitis, where it acts in the vicinity of nerve fibres and therefore has a nerve-targeting effect, which is not the case in pemphigus.

In PH, the mechanism by which IgG autoantibodies produce the characteristic skin lesions of PH (28, 50) is still debated, and the target antigens are usually Dsg1 and less commonly Dsg3. Non-Dsg cases may occur. According to Karray and coworkers (50), the intense inflammation may not be associated with acantholysis and PH has a broader epitope distribution compared to PV and PF. Proinflammatory cytokines released from activated keratinocytes include IL-8, which is involved in eosinophil and neutrophil chemotactic activity. Eosinophilia in the peripheral blood has been reported in some patients and may be associated with pruritus (28).

In IgA pemphigus, pruritus is an expression of IgA antibodies specifically directed against Dsgl-1, Dsgl3, Dsc-1. The precise immune cascade of IgA pemphigus remains elusive (28), but it is certain that PMNs infiltrate the integument, which is why the disease is often classified as a neutrophilic dermatosis.

## Hallmarks and challenges in different types of pemphigus

### Pemphigus herpetiformis

**Figure 8:**
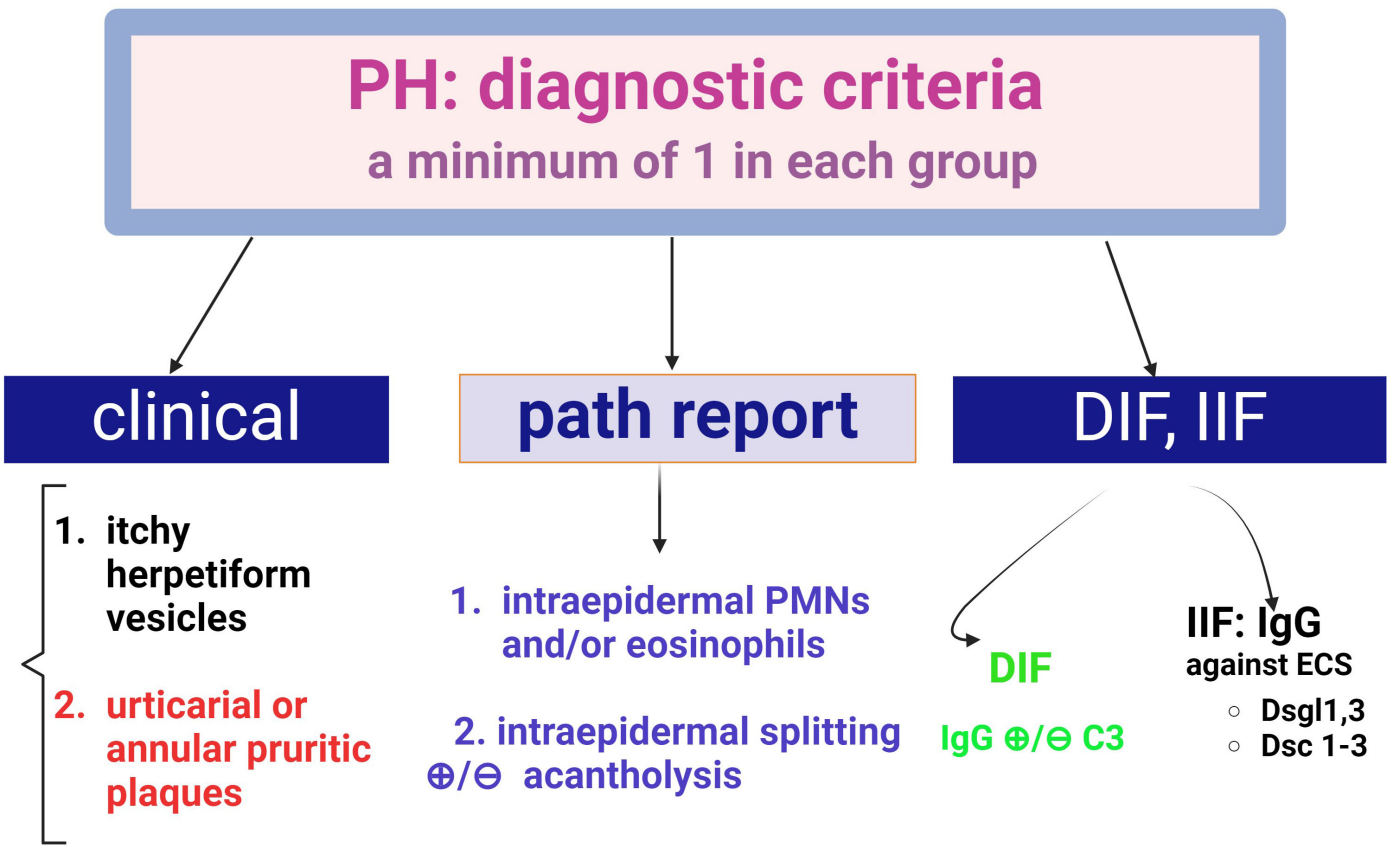
Diagnostic criteria for Pemphigus Herpetiformis (PH) (adapted from Kosta LMC.; Cappel M.A.; Keeling J.H, 2019, (47), There are three diagnostic groups- clinical, histopathological and (direct and indirect) fluorescence criteria. A positive diagnosis requires at least one criterion from each group.

Progress has been made in the diagnosis of this form of pemphigus with the introduction of diagnostic criteria for PH (**Figure 8**) (51). The presence of pruritus, the herpetiform configuration of the lesions and the sparring of the mucosa are clinical features that need to be reinforced by the presence of IgG deposits in DIF.

Despite the name “herpetiform”, it has nothing to do with a viral infection. The same confusion may occur with dermatitis “herpetiformis” (Dühring-Brocq disease) or pemphigoid *gravidorum* (“*herpes gestationis*”).

The intense pruritus and vesiculo-bullous exanthema (**Figure 8**) make clinical differentiation from a dermatitis herpetiformis or bullous pemphigoid difficult, but the presence of acantholysis and DIFpositivity (IgG) improves diagnostic accuracy. In other words, pemphigus herpetiformis may share clinical features with dermatitis herpetiformis and immunological features with immune pemphigus (51). Occasionally, autoantibodies are negative in pemphigus herpetiformis.

The variety of situations in which pemphigus herpetiformis migrates (both clinically and para-clinically) into either pemphigus foliaceus or pemphigus vulgaris is a diagnostic challenge that requires the expertise of the clinician (52–54).

### Senear-Usher pemphigus and bullous lupus: the difficult differential diagnosis

**Figure 9:**
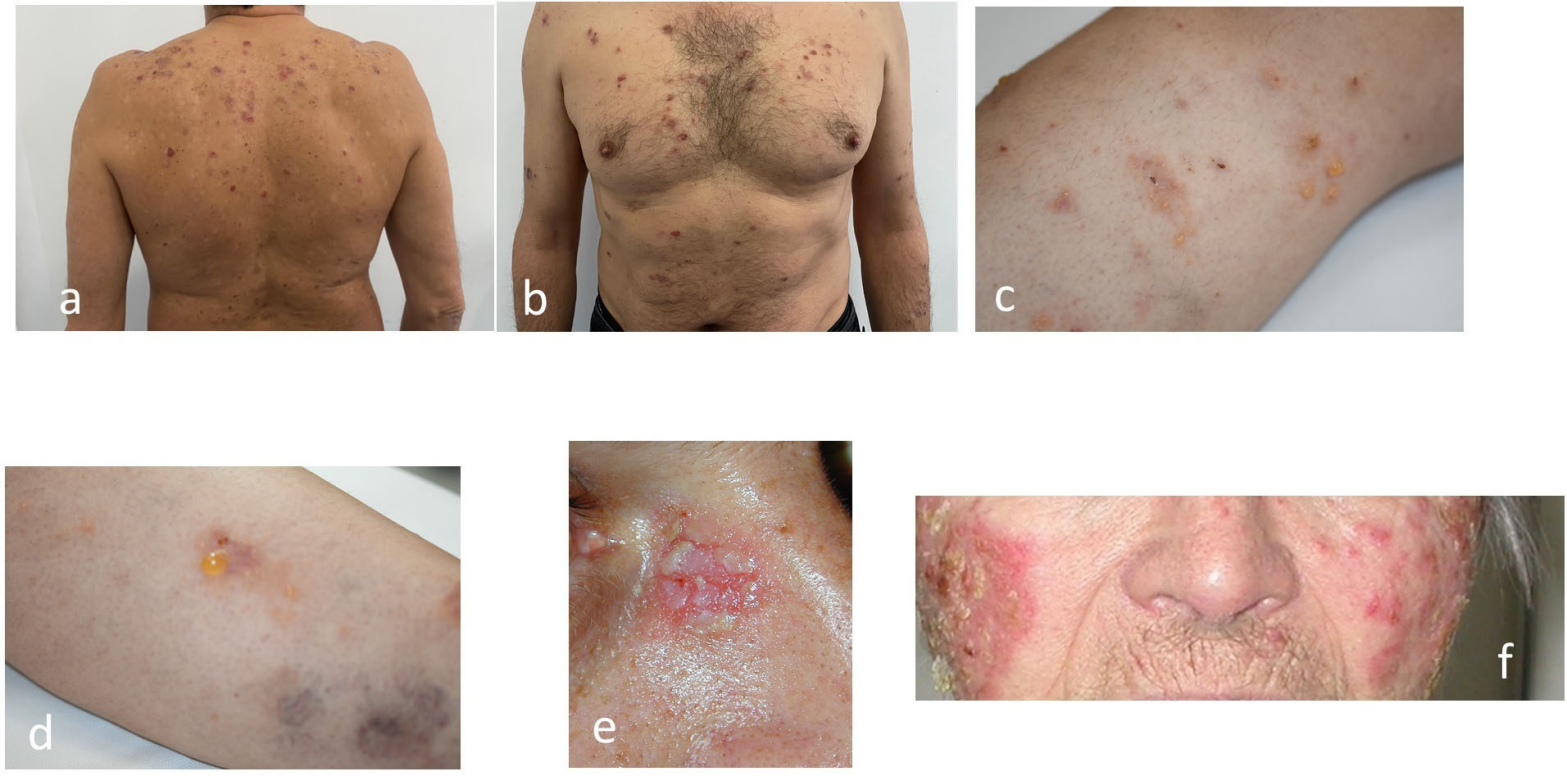
Pemphigus superficialis: **(a, b)** Pemphigus Herpetiformis (PH) **(c, d)**. IgA pemphigus **(e, f)**. Pemphigus erythematosus. In the erythematous form, note the photodistribution of the lesions. (authors’ collection cases).

Almost 6 decades after the initial description of photodistributed pemphigus erythematosus (PE) by Senear and Usher, Weston (1981) and Sontheimer (1979, 1982) described anti-Ro (SSA) and anti-La antibodies and their relationship to subacute lupus erythematosus. This type of Senear-Usher pemphigus is a superficial pemphigus (**Figure 9**) that overlaps the features of lupus erythematosus, sometimes with these autoantibodies and/or dsDNA being positive. Some explanations correlate the coexistence of antiepithelial and antinuclear specificities (55) in PE.

In terms of clinical presentation, the bullous exanthema of PE affects the seborrhoeic areas and scalp, with “brain-like” lesions tending to crust (33, 56). Bullous systemic lupus erythematosus affects more women than men and the lesions are photodistributed (55, 56).

Histopathological examination provides elements of certainty according to the location of the bulla: subepidermal in bullous lupus (non-achantolytic) and intraepidermal (achantolytic) in Senear-Usher Pemphigus (44). DIF is also accurate and shows both the classic pemphigus focal deposits of IgG within the intercellular space (57) of the epidermis and the “lupic band” at the dermoepidermal junction (granular deposits of IgG and IgM) in Senear-Usher pemphigus. Based on the authors’ DIF experience, the epiluminescence microscopist may encounter this appearance on the same or different slides from the same patient. In patients with bullous lupus, linear or granular deposits (IgG, M, A, complement) along the dermo-epidermal junction are seen on DIF (56) due to autoantibodies directed against type VII collagen.

### Special features in IgA pemphigus

As a “neutrophilic dermatosis”, IgA pemphigus is a diagnostic challenge (58), and differentiation from Grover’s transient acantholytic dermatosis, Darier’s follicular dyskeratosis and Sweet’s neutrophilic dermatosis, is often difficult. Furthermore, even distinguishing between the two forms, subcorneal pustular dermatosis (SPD) and intraepidermal neutrophilic type (IEN type), requires an experienced team, dermatopathologist and dermatologist (58–60). The site of acantholysis in SPD is subcorneal, whereas in IEN it is inferior, i.e. suprabasal (44, 61).

The targets of IgA autoantibodies in SPD type are desmosomal cadherins (Dsc1, Dsc2, Dsc3), whereas no major autoantigenic profile has been identified in IEN type (62), suggesting that its autoantigenic profile is heterogeneous. Dsg are recognised by IgA antibodies in a few patients with IgA pemphigus (62). Intraepidermal proteolytic cleavage leading to acantholysis is mediated by PMNs, which accumulate with monocytes as a result of possible binding of IgA autoantibodies to the Fc receptor CD89.

Clinically, both forms of pemphigus IgA are associated with a pruritic, vesiculo-pustular exanthem (59) localised on the trunk and extremities, often confluent, with vesicles that rupture and crust centrally (**Figure 9**). The intraepidermal neutrophilic variant has a characteristic configuration known as the “sunflower” (60, 61). In paediatric patients, the main differential diagnosis is the dermatosis with linear deposition of IgA. DIF resolves the problem with intraepidermal fluorescence in IgA pemphigus and linear basement membrane fluorescence in linear IgA dermatosis. Involvement of intertriginous areas raises differential diagnostic issues (see above).

We note the association of IgA pemphigus with monoclonal gammopathy (63), HIV infection (64), inflammatory bowel disease (65), rheumatoid arthritis (66), drug administration (immune checkpoint (67), inhibitors or thiol drugs) (68).

Recently, a new form of IgG/IgA pemphigus has been described, the clinicopathological features of which differ from those of classical IgG and IgA pemphigus (69, 70). IgG/IgA pemphigus is defined by the presence of IgG and IgA cell surface deposits on DIF and/or circulating IgG and IgA autoantibodies on IIF (70). The few case reports suggest that IgG/IgA pemphigus resembles IgG pemphigus in clinical features, IFD and IIF, but differs significantly from IgA patients in intertriginous distribution, pustular lesions, achantolysis and DIF (68, 69). Thus, Lehman and co-workers have suggested that IgG/IgA pemphigus may be a variant of IgG pemphigus rather than an IgA pemphigus (69, 70).

Another problem is the relationship between IgA superficial pemphigus (SPD) and Sneddon-Wilkinson subcorneal pustulosis, SWD, which is still considered a “spectrum of disease” (71).

Subcorneal pustular dermatosis, described by Ian Sneddon and Darrell Wilkinson in 1956, is a neutrophilic dermatosis and it is still unclear whether it is distinct from IgA pemphigus or is part of a spectrum of the same disease (72). IgA pemphigus was described 26 years later by Wallach, Foldes, and Cottenot as “subcorneal pustular dermatosis and monoclonal IgA”. IgA pemphigus is distinguished from SWD by a positive DIF (**Figure 4**) showing intercellular IgA deposition (71, 73). Other authors define SWD as a “benign amicrobial pustulosis” that belongs to the spectrum of neutrophilic dermatoses and may be associated with IgA monoclonal gammopathy and other neutrophilic dermatoses (71).

### Distinctive features of drug-induced pemphigus

In this rare form of pemphigus, there is no consensus on the mechanism of pathogenesis, but an interaction between genetic predisposition (HLA-DRB1) and environmental factors has been documented (74, 75). Acantholysis occurs as a result of two distinct processes: biochemical interactions at the basement membrane of keratinocytes (non-immune) and (intracellular) anti-Dsg1 and/or Dsg3 (immune) antibodies (76).

The offending chemical groups are *thiol drugs* (penicillamine, HLA-B15 predisposition), *phenol group drugs* (aspirin, rifampicin, anticonvulsant cephalosporins) and *non-thiol/non-phenol drugs* (converting enzyme inhibitors, checkpoint inhibitors) (75, 76).

Thiol drugs contain a sulfhydryl group (-SH) and are the most common drugs that induce pemphigus by biochemical modification of the antigen (74, 76). Three mechanisms have been correlated with achantholysis in thiol-containing drugs: neo-epitopes *via* interaction with Dsg1 and 3, alteration of the basal membrane *via* the keratinocyte (disulfide bond) and the pathway of proteolytic enzymes (plasmin) (75).

The phenolic group (-OH) acts at the level of the keratinocytes, which release TNF-α and IL-1, followed by activation of plasminogen and other proteases (74).

Non-thiol/non-phenolic drugs act through antibody formation. New immunological therapies such as checkpoint inhibitors are opening the door to cutaneous autoimmune diseases. Although checkpoint inhibitors are known to induce bullous pemphigoid (77), there are already reports that the combination of ipilimumab plus nivolumab can induce PV as an immune-related adverse event (3). It is important to note that pemphigus improves with drug withdrawal. Most reports of D-penicillamine-induced pemphigus vulgaris have been described in patients with rheumatoid arthritis (78). The question has been raised as to whether the treatment induces pemphigus or represents an association of the disease with rheumatoid arthritis, as pemphigus vulgaris has been described in patients with rheumatoid arthritis who are not taking penicillamine (78).

### Pregnancy and pemphigus

Pemphigoid (herpes) *gestationis/gravidorum* is an exclusive bullous dermatosis of pregnancy (79–81), but other immune bullous diseases may be found or worsen during pregnancy. It is not only difficult to treat but also to diagnose (81).

PV may worsen during pregnancy (81), especially in the first two trimesters, due to the Th2 predominance, in addition to the hormonal influence (oestrogen, progesterone, cortisol). In the third trimester, the chorion itself produces corticoids (endogenous) that can suppress autoimmune responses. The condition, which is fortunately rare, is associated with a risk of prematurity, infant death and pemphigus *neonatorum*, but the baby may be born healthy. Neonatal pemphigus may be due to transplacental transfer of IgG4 autoantibodies.

The term “syphilitic pemphigus” refers to congenital syphilis (82) (e.g. *Treponema pallidum* infection) and has nothing to do with “pemphigus”. The term derives from the fact that all bullous diseases were originally called “pemphigus”.

### Paraneoplastic pemphigus revisited

Described by Anhalt and coworkers in 1990 (83), PNP is associated with established or occult malignancies (37, 41, 84, 85), mainly haematological: non-Hodgkin’s lymphoma (86), chronic lymphocytic leukaemia (CLL) (87), Castleman’s disease (**Figures 4**, **7**) (88), thymoma, and Waldenstrom’s macroglobulinemia (89). Clinically (41, 44, 45), the enanthema is severe, intractable (37, 41), with lesions around the mouth and nose. The exanthema also has erythema multiform-like lesions, which must be distinguished from other forms of pemphigus or even bullous pemphigoid. The authors have treated PNP cases in which the skin lesions were so extensive that the confluence of the plaques and the rupture of the bullae produced a TEN/Lyell-like appearance. In addition to skin lesions, damage to the respiratory epithelium can lead to death (90).

The term *paraneoplastic autoimmune multi-organ syndrome* (PAMS), classically described as PNP by Anhalt et al., indicates the severity of multisystem involvement (90).

**Figure 10:**
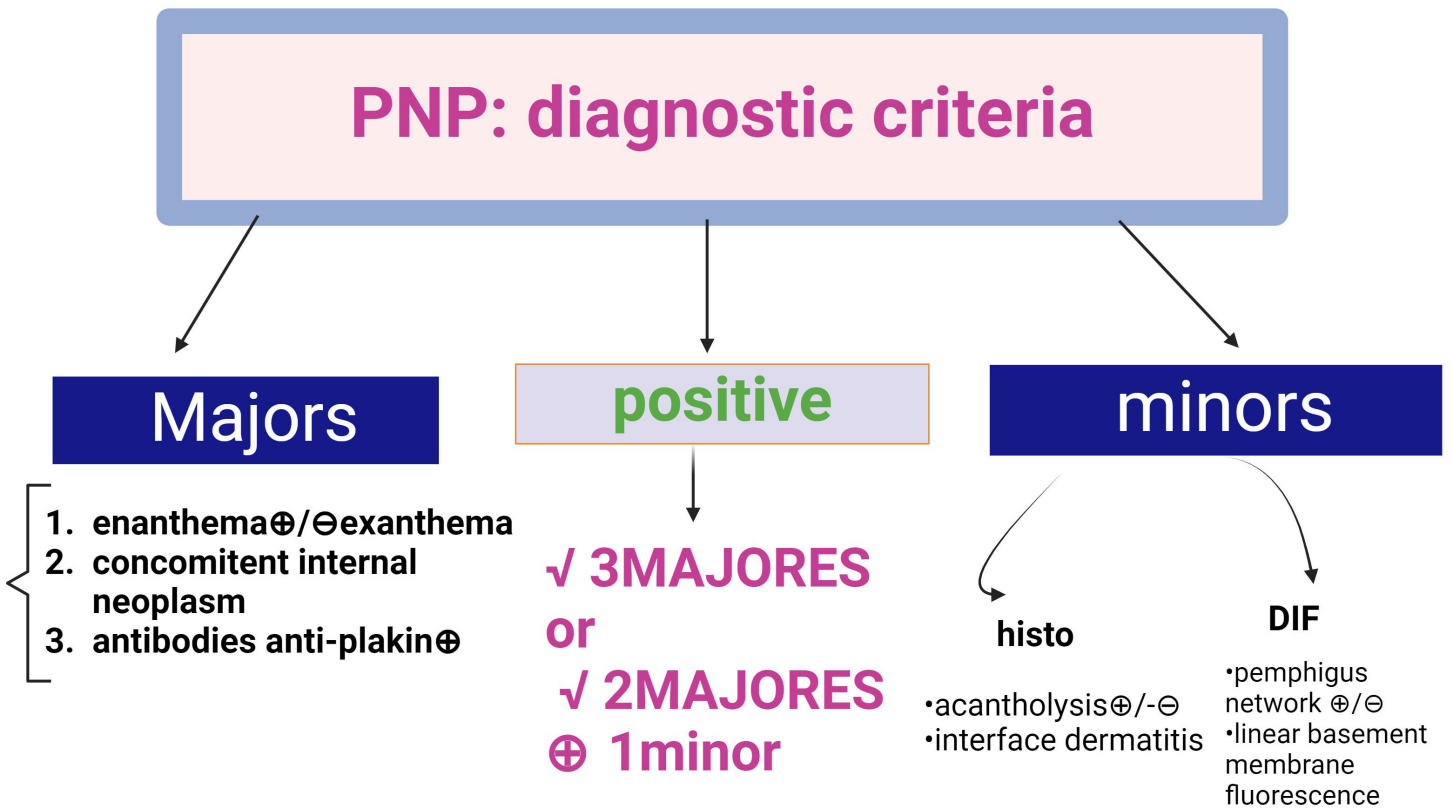
Diagnostic criteria for Paraneoplastic Pemphigus (PNP) (adapted from EADV guideline 2023 and Svoboda (89, 90). There are two categories of criteria, major and minor. A positive diagnosis fulfils 3 major criteria or 2 major + 1 minor criteria.

Histopathological criteria include interface dermatitis and acantholysis throughout the epidermis (91). Ig G-type autoantibodies are directed against cadherin (Dsg1,3) but also against non-cadherin antigens (evoplakin, periplakin, BP1, 2 Ag and A2ML protein) (13, 85, 92). This explains why the appearance of DIF shows intercellular intraepidermal fluorescence, but sometimes an appearance suggestive of bullous pemphigoid (fine, linear and continuous basement membrane fluorescence in IgG autoantibodies). The diagnosis is supported by the new 2023 criteria (91, 92) (**Figure 10**).

### Can pemphigus subtypes cross over?

In eosinophilic spongiosis and pemphigus herpetiformis (52, 53), there is strong evidence for transition to other forms of pemphigus. Since 2002, cases of transition between PH and PF (52), PV vulgaris and PF (54, 93–95), naively or after treatment with rituximab (52, 53), have been reported. Transitions from PF to PV (53), PH to other forms, and eosinophilic spongiosis to PV or PF have also been documented. The topic is still controversial and the evidence for the rare cases so far is represented by anti-Dsgl ELISA autoantibodies.

The mechanism of the transition between PV and PF remains elusive (94) and the phenomenon of *epitope* sp*reading* has been suggested. There are two types of immune response involved: a primary or inflammatory autoimmune response, which causes tissue damage by exposing the body’s immune system to a protein that evades immunological detection by the immune system, thereby triggering a secondary autoimmune response. Amino-terminal pathogenic antibodies to the EC domain of Dsg1 have been reported to be maintained during the transition from PV to PF, whereas significant epitope changes occurred in response to Dsg3, with an absolute or significant decrease in pathogenic antibodies to the EC1 domain of Dsg3 (95). The transition is associated with a decrease in anti-Dsg3 autoantibodies and an increase in Dsg1.

In terms of clinical presentation and prognostic value, autoantibody changes may be beneficial for a patient progressing from PV to the superficial forms. Some authors disagree with the shift between different forms of pemphigus, as recent reports of IgG/IgA pemphigus show heterogeneous clinical and histopathological presentations (68).

### Does autoimmune pemphigus have a genetic component?

**Figure 11:**
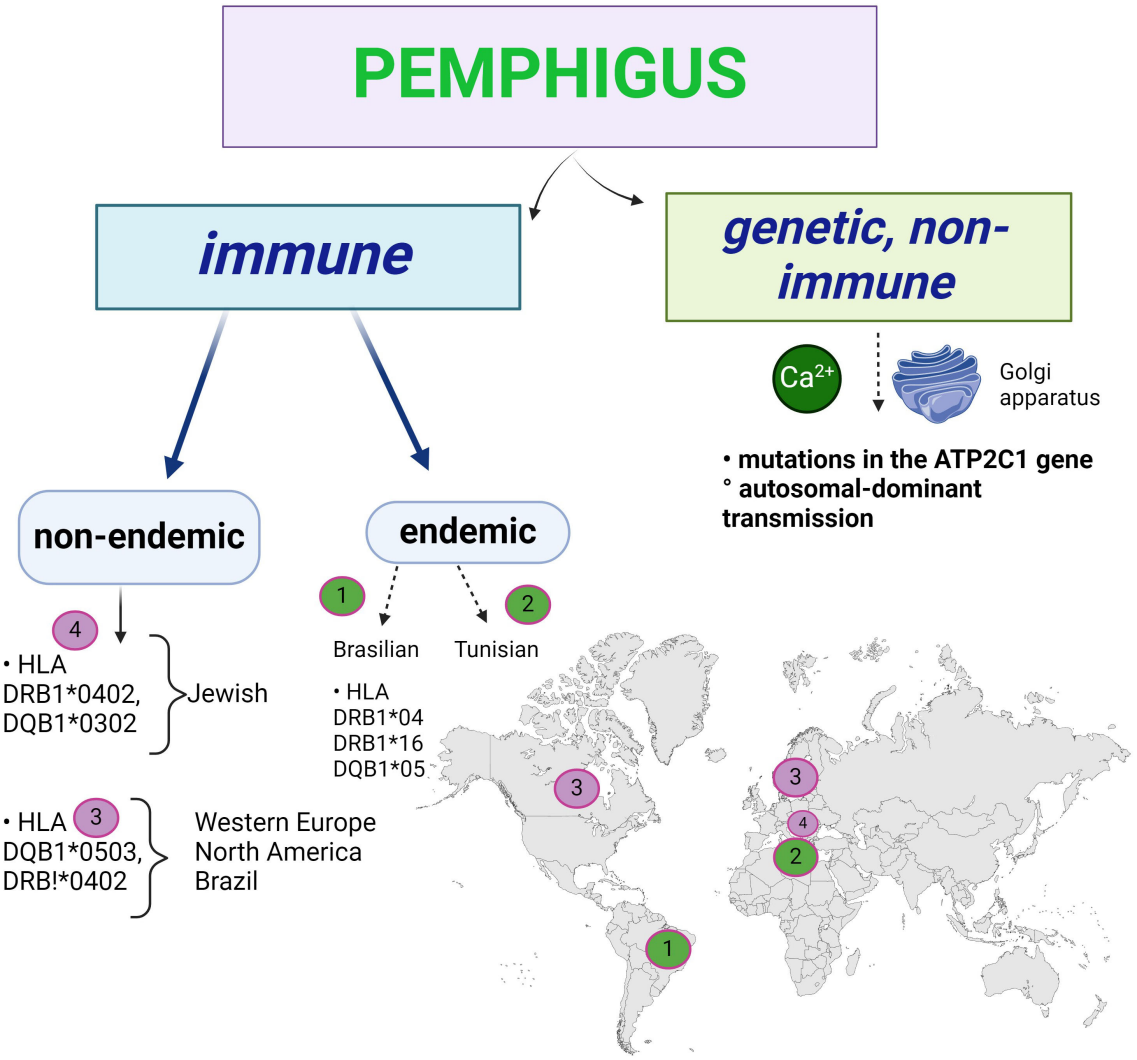
The genetic basis of pemphigus: Hailey-Hailey familial benign pemphigus is an autosomal dominant genetic disorder involving mutations in the ATP2C1 gene. Immune pemphigus is not a genetic disease, but there are populations with HLA predisposition.

Genetically, there are two categories of pemphigus: familial genetic (Hailey-Hailey) and non-genetic, immunological (**Figure 11**). The latter has an endemic form (Brazilian and Tunisian) and a non-endemic form.

The genetic form of acantholysis is non-immunological, the result of an autosomal dominant inheritance, and the chromosome involved is chromosome 3, with a mutation on its long arm (96). The ATP2C1 gene, which codes for the ATP-dependent Ca^2+^ and Mn^2+^ transporter, is mutated in such a way that the non-immunological acantholysis is related to Ca^2+^ at the Golgi apparatus of the keratinocytes.

Acantholysis is immunological in the non-genetic form as shown above. In the immunologically endemic Brazilian pemphigus (fogo selvagem), HLA class II DRB1*04, DRB1*16, DQB1*05 predominate (97) (**Figure 11**). The endemic forms of pemphigus (Brazilian and North African, Tunisian) take into account the area of distribution of the disease, which has a multifactorial aetiology, in this case a familial predisposition. Salivary antigens from *Simulium pruinosum* (“black fly”) induce a cross-reaction leading to the production of autoantibodies against DSG1 in Brazilian pemphigus (20), where the disease occurs in members of the same family living near water. It is not clear why the incidence of the disease is lower in the northern regions where these insects are found, so other viruses or micro-organisms may be involved. Apoptosis is being studied, but other cell death has not been investigated, but mitochondrial autoantigens are acetylcholine receptors (20).

In immunologically non-endemic pemphigus there are associations with HLA Ag class II (DRB1*0402, DQB1*0302- in Jews) (98) and DQB1*0503, DRB1*0402 (Western Europe, North America and Brazil). There are numerous ethnic and geographical variations, not including endemic pemphigus, with high prevalence described in Ashkenazi Jews and Mediterranean populations (33). ST18 gene mutations confer a 6-fold increased risk of developing pemphigus vulgaris compared to the general population, via TNF-alpha, whose integumentary expression is increased (99, 100). A polymorphism of Dsg3 has been observed in association with pemphigus cls II susceptibility alleles, which may contribute to the development of PV (98). Although several attempts have been made to identify susceptibility traits, our knowledge of the genetic basis of PV is far from complete (99).

As shown above, an interaction between genetic predisposition (HLA-DRB1) and environmental factors has been documented in post-medication pemphigus.

More research is needed to understand the role of HLA molecules in immune pemphigus (98), mainly in the treatment, whereas amazing progress has been made in characterising them in psoriasis, where they correlate with different types of disease.

## Gold standard for diagnosing and evaluating immune pemphigus

### DIF in the diagnosis of immune pemphigus: its role and limitations

The place of DIF (**Figure 4**) in the diagnostic staging of pemphigus forms is immediately after the histopathological examination in HE staining, which identifies the site of acantholysis and describes for each bulla: the ceiling, the floor and the contents (fluid in which the acantholytic cells float). “Spongiosis with eosinophils” is a histopathological feature characteristic of early pemphigus that the clinician should be aware of for two reasons: it is also seen at the onset of PB and, as mentioned above, it can be seen in other forms of pemphigus (28, 53).

DIF is an important diagnostic test (34, 101–103) in immunological pemphigus. The sensitivity of DIF is 94-98% (103, 104) with a positive predictive value of 90%, but a specificity of 36.3%. DIF is an accurate tool for confirming a definitive diagnosis (34, 102). Ideally, the dermatopathologist should note the concordance between routine HE and DIF.

The sampling technique is important: punch biopsies containing the bulla and perilesional tegument (3-10mm (34) are preferred on the skin, a formulation that also provides the necessary depth. This allows us to differentiate epidermal vesicles/bullae from deeper ones. In the mucosa, bleeding and local access complicate sampling. As mentioned above, ideally, two fragments should be obtained: the lesional tegument and the mucosal tissue (34).

If the site of acantholysis is important for histopathology, the DIF result will be positive or negative, indicating (**Figure 4**): fluorescence of the stratum corneum (correct execution marker), intraepidermal immunoglobulin (Ig)G antibodies or C3 on the cell surfaces of keratinocytes (reticular intercellular IgG, IgA, C3). The site of acantholysis is not noted, only the fluorescent aspect. This pattern of deposition of IgG and C3 in intercellular space staining (ICS) has been termed “chicken wire” or “fish net” appearance. A positive DIF is one of the markers of PV relapse and is useful in monitoring the disease. In IgA pemphigus, fluorescence is superficial in SPD (restricted to the upper epidermal cell surfaces) and deeper in IEN (intercellular IgA deposition restricted to the lower epidermis or throughout the epidermis) (105–107). As mentioned above, these targets of IgA autoantibodies in SPD are desmosomal cadherins (Dsc1-3) (61).

The above mentioned DIF appearance in PNP is due to the attack initiated by both autoantibodies and CD8+ lymphocytes: intercellular and basement membrane deposition of IgG and C3, leading to confusion even with bullous pemphigoid. Interactions between neoplastic antigens and autoantibodies cross-reacting with epithelial antigens have been suggested. Another theory focuses on proinflammatory cytokines (IL-6) produced by autoantibodies synthesised by cancerous tumours. The presence of IgG and/or C3 in the ICS and in the basement membrane zone (BMZ) has been reported in less than 50% of cases. This is probably due to the expression of the bullous pemphigoid antigens BP1Ag and BP2Ag or the involvement of CD8+ lymphocytes in the initial attack (108).

In Senear-Usher erythematous pemphigus, a mixed pattern is seen on the slide or on different slides of the same specimen: a network in IgG and/or C3, as well as banded deposits at the dermal-epidermal junction, a “band-like” appearance, overlapping with pemphigus-erythematous lupus (58).

If DIF is negative on histopathological examination with acantholysis, the pemphigus is non-immunological (Hailey-Hailey or drug-induced non-immune form) or the case is treated with immunological remission. DIF may also be negative if the patient is receiving immunosuppressive treatment at the time of sampling (108), or if sampling and transport errors occur. If the DIF is *false positive*, a false reticulated appearance will be obtained due to technical deficiencies (freezing, crushing of the sample).

In situations where DIF is *not available* due to lack of epiluminescence microscope, staff training or reagents, the method could be replaced by IHC tests for IgG, C3d, C4 with efficient diagnostic reports. IHC on FFPE sections can be used as an alternative method to DIF (109). For example, a study of 20 patients with pemphigus vulgaris showed 85% positivity for IgG and 95% positivity for C3 on IHC, and 6 patients with pemphigus foliaceus showed 100% positivity for IgG and 75% positivity for C3 (109). Other authors have evaluated IHC for C4d in patients with immunological pemphigus and bullous pemphigoid. They found C4d deposits on intraepidermal DIF in 77.2% of patients, and the sensitivity of this technique was higher than that of classical serological techniques. This led to the suggestion that IHC could be considered in cases where serological tests were available (110).

### Additional serological tests and their value

These tests are minimally invasive, but their role is complementary and their value is indicative. They cannot replace the combination of histo-pathology and DIF (111), so their role is to increase diagnostic accuracy. Serological tests are mainly based on IIF and ELISA and detect circulating autoantibodies (112, 113).

IIF detects IgG and IgG4 autoantibodies (96, 113) on various substrates containing Dsg1 and Dsgl3. These substrates are monkey oesophagus (112), guinea pig oesophageal epithelium, normal human skin (incubated with NaCl to separate the epidermis from the dermis). The greater the number of substrates, the greater the accuracy of the method. The IIF substrate is basically a mosaic with 6 detection zones which can identify both autoantibodies against pemphigus and those directed against the BP1Ag and BP2Ag. Other types of substrate for IIF, such as primate liver tissue (useful for dermatitis herpetiformis), are not used for pemphigus. IIF antibody titre correlates with disease activity (112). A marker of PV relapse is positivity for antibodies to Dsg3.

The ELISA test is a sensitive diagnostic tool (112) that detects antibodies to Dsg 1, Dsg 3, -evoplakin, -collagen VII and BP-180, 230. It is useful for both screening and monitoring of pemphigus. In clinical practice, the multistep approach (112, 113) is recommended, with IIF screening followed by ELISA. The earlier this is done, the more reliable the diagnosis.

IgA pemphigus is serologically negative for Dsg1,3 and IgA positive for Dsc. In PH, serology is a diagnostic criterion with positive Dsg-1 antibodies (rarely Dsg3), Dsc 1,3 and an unknown 178kDa protein (16, 19).

Other serological tests include *immunoblotting* and *immunoprecipitation*, which detect rare autoantibodies (113, 114) (anti-laminin gamma 1, anti-laminin 332, anti-LAD-1, anti-alpha6, beta integrins, anti-Dsp, collagen VII). They are based on recombinant proteins or cell extracts and are used for screening rather than diagnosis. They require specialised laboratories and trained personnel and are laborious and time-consuming.

As shown above, PNP is associated with autoantibodies directed against cadherins, but also against periplakin, envoplakin, BP 1 and 2 antigens, and α2-macroglobulin-like-1 (A2ML1) protein. Drug-induced pemphigus involves Dsg 1 and 3, and IgA predominantly Dsg-1 (subcorneal pustulosis) or Dsg 1 and 3 in the deeper forms.

There are numerous case reports of individuals with positive antibodies to Dsg1 and Dsg3 antigens without a pemphigus phenotype. A 2018 study (115) found that almost half of subjects in a population in Amazonian Peru had positive anti-Dsg1 antibodies and approximately 25% had positive anti-Dsg3 antibodies in the absence of pemphigus lesions, highlighting the role of environmental and ethnographic factors.

Similarly, anti-Dsg antibodies have been reported in patients with inflammatory dermatological disorders: *pyoderma gangrenosum*, erythema multiforme major, atopic dermatitis, lichen planus pemphigoid, BP (116) or following viral infections or vaccination (117, 118).

## Discussion

Recently recognised as a desmosomal disorder, autoimmune pemphigus remains severe in some of its forms (e.g. PV). Therefore, an accurate diagnosis allows for correct treatment, which is all the more effective the earlier it is started.

Advances in the understanding of antigens, the discovery of new forms of pemphigus and the introduction of updated criteria for the diagnosis of PNP and PH facilitate this diagnosis, but the presence of pruritus in some forms of pemphigus is a recent achievement, as is the entity of PH. At the molecular level, the role of cytoplasmic organelles, the involvement of keratinocytes, lymphocytes, eosinophils and PMNs in cadherins that induce acantholysis/apoptolysis is becoming increasingly clear.

Acantholysis, DIF and serological testing remain the diagnostic pillars. The identification of proteins other than Dsgl has made it possible to understand the DIF aspect of PNP. IHC can replace IFD when it is not available. Progress in understanding the DIF aspect of PNP and PF (Senear-Usher) has resulted from superior characterisation of antigenic targets and represents a major achievement today. Important advances in multi-substrate laboratory diagnostics in autoantibody detection by IIF combined with ELISA are based on the increasingly articulated nature of cadherins and their different mechanisms of action in various forms of pemphigus.

Clinical diagnostic clues (enanthema, intertrigo, pruritus, distribution of lesions, standardised diagnostic criteria in PNP and PH) and specific and difficult to differentiate situations between bullous lupus and Senear-Usher autoimmune pemphigus, between forms of IgA pemphigus or differentiation with other autoimmune diseases or neutrophilic dermatoses are presented. Today, specialised pemphigus clinics exist in some tertiary services around the world, but in most countries the patient is referred to the general dermatologist, who has to make the best therapeutic decision, often on an emergency basis, using a set of criteria for diagnosis and differentiation between the clinical forms of bullous diseases, one of the most difficult chapters in dermatology.

Eosinophilic spongiosis is an early histo-pathological feature and requires an experienced dermatopathologist and clinician who is aware that it can cross over into any form of immune pemphigus, but also that it is part of the diagnostic criteria for PH. Crossover between different forms of pemphigus is rare and controversial.

Drug-induced pemphigus is gaining new importance in the era of immunological therapies for cancer. The classical aetiology (thiol, non-thiol or phenol groups) has been joined by the recent class of checkpoint inhibitors, which are now widely used in oncology. In the specific case of pregnant women, two issues arise: the analysis of the diagnosis of pemphigus in pregnancy, where pemphigoid *gravidorum* is an exclusive bullous dermatosis of pregnancy, and the delicate clinical analysis (laboratory-based) of the worsening of immune pemphigus in the first two trimesters.

Last but not least, we are no longer afraid to discuss genetic predisposition in autoimmune pemphigus, classically considered a non-genetic disease. The existence of endemic forms of the disease and the distribution of HLA molecules in different populations and in patients with different comorbidities are steps forward in establishing the genetic component of immune pemphigus. Further research is needed to help characterise it and to catch up with progress made in other diseases (e.g. psoriasis, lupus erythematosus).

The present review is the first to combine difficulties in clinical diagnosis with new molecular insights. It provides a comprehensive overview of recent advances in the understanding of autoimmune pemphigus, bridging the clinical challenges and complexities of diagnosing different forms of pemphigus with new molecular insights, and providing a valuable resource for clinicians caring for patients with pemphigus.

## Glossary

PV: pemphigus vulgaris
HLA: Human leukocyte antigen
PMNs: polymorphonuclear neutrophils
PNP: paraneoplastic pemphigus
PH: pemphigus herpetiformis
Dsg: desmoglein
Dsc: desmocolline
Pg: plakoglobin
Pkp: plakophilins
Dsp: desmoplakin
c-Myc: proto-oncogene, cellular-multifunctional transcription factor
p38MAPK: p38 mitogen-activated protein kinases
RhoA: Ras homolog family member A
PF: pemphigus foliaceous
PLC: phospholipase C
IgG: immunoglobulin G
IgA: immunoglobulin A
IgM: immunoglobulin M
Th: T helper cell
IL: interleukin
Treg: T cell regulatory
IFN-γ: interferon γ
NKTcells: natural killer T lymphocytes
TLO: tertiary lymphoid organ
BP: bullous pemphigoid
BP2Ag: bullous pemphigoid antigen 2, 180 Kdal
BP1Ag: bullous pemphigoid antigen1, 230 Kdal.
p38: protein 38, the “guardian of the genome”
MAPK: mitogen-activated protein kinase
EGFR: epidermal growth factor receptor
ERK: extracellular signal-regulated kinase
c-Jun: transcription factor, (PLC)γ1, phospholipase C γ1
Fas-ligand: membrane protein
LGI1: leucine-rich glioma inactivated 1 antibody
NCCD: Nomenclature Committee on Cell Death
CASPR 2: contactin-associated protein-like 2
FcΔRIIB: inhibitory Fc receptor B
SPD: subcorneal pustular dermatosis
AMA: antimitochondrial antibody
SER: smooth endoplasmic reticulum
SPCA1: ATPase pump type 1
DIF: direct immunofluorescence
C3: complement component 3
IIF: indirect immunofluorescence
ELISA: enzyme linked immunosorbent assay
BP 1 Ag: bullous pemphigoid antigen 1, 230 KDal
Pk: plakins
A2ML1: α-2-macroglobulin-like 1
PH: pemphigus herpetiformis
IEN: intraepidermal neutrophilic type
SWD: Sneddon-Wilkinson subcorneal pustulosis
HLA: Human leukocyte antigen
-SH: Sulfhydryl group
-OH: phenolic group
TNF-α: tumour necrosis factor α
MBZ: membrane basal zone
CLL: chronic lymphocytic leukaemia
PAMS: paraneoplastic autoimmune multi-organ syndrome
ICS: intercellular space staining
IHC: immunohistochemistry
FFPE: formalin-fixed paraffin-embedded
C4d: complement 4d
ECS: epithelial cell surface

## References

[1] Hutchison DM Hosking AM Hong EM Grando SA. Mitochondrial autoantibodies and the role of apoptosis in pemphigus vulgaris. Antibodies (Basel). (2022) 11:55. doi: 10.3390/antib11030055 36134951 PMC9495650

[2] Yeruva S Waschke J. Structure and regulation of desmosomes in intercalated discs: Lessons from epithelia. J Anat. (2023) 242:81–90. doi: 10.1111/joa.13634 35128661 PMC9773171

[3] Nakamura H Shionoya A Arihara Y Hayasaka N Kubo T Usami M. Pemphigus vulgaris as an immune-related adverse event in recurrent metastatic esophageal squamous cell carcinoma treated with ipilimumab plus nivolumab: a case report and literature review. Front Immunol. (2023) 14:1259071. doi: 10.3389/fimmu.2023.1259071 37753079 PMC10518453

[4] Micaroni M Giacchetti G Plebani R Xiao GG Federici L. ATP2C1 gene mutations in Hailey-Hailey disease and possible roles of SPCA1 isoforms in membrane trafficking. Cell Death Dis. (2016) 7:e2259. doi: 10.1038/cddis.2016.147 27277681 PMC5143377

[5] Schmitt T Egu DT Walter E Sigmund AM Eichkorn R Yazdi A. Ca^2+^ signalling is critical for autoantibody-induced blistering of human epidermis in pemphigus. Br J Dermatol. (2021) 185:595–604. doi: 10.1111/bjd.20091 33792909

[6] Perez TD Nelson WJ. Cadherin adhesion: mechanisms and molecular interactions. Handb Exp Pharmacol. (2004) 165):3–21. doi: 10.1007/978-3-540-68170-0_1 PMC3368609 20455088

[7] Egu DT Schmitt T Waschke J. Mechanisms causing acantholysis in pemphigus-lessons from human skin. Front Immunol. (2022) 13:884067. doi: 10.3389/fimmu.2022.884067 35720332 PMC9205406

[8] Kim SA Tai CY Mok LP Mosser EA Schuman EM. Calcium-dependent dynamics of cadherin interactions at cell-cell junctions. Proc Natl Acad Sci U S A. (2011) 108:9857–62. doi: 10.1073/pnas.1019003108 PMC3116393 21613566

[9] Ishii N. Significance of anti-desmocollin autoantibodies in pemphigus. J Dermatol. (2023) 50:132–9. doi: 10.1111/1346-8138.16660 PMC10107560 36578135

[10] Maître JL Heisenberg CP. Three functions of cadherins in cell adhesion. Curr Biol. (2013) 23:R626–33. doi: 10.1016/j.jdermsci.2007.05.005 PMC3722483 23885883

[11] Sharma P Mao X Payne AS. Beyond steric hindrance: the role of adhesion signaling pathways in the pathogenesis of pemphigus. J Dermatol Sci. (2007) 48:1–14. doi: 10.1016/j.jdermsci.2007.05.005. Epub 2007 Jun 18. PMID: 17574391 17574391

[12] Steinert L Fuchs M Sigmund AM Didona D Hudemann C Möbs C. Desmosomal hyper-adhesion affects direct inhibition of desmoglein interactions in pemphigus. J Invest Dermatol. (2024) 144(12):2628–94.e10. doi: 10.1016/jid.2024.03.042 38677661

[13] Schmitt T Pircher J Steinert L Meier K Ghoreschi K Vielmuth F. Dsg1 and dsg3 composition of desmosomes across human epidermis and alterations in pemphigus vulgaris patient skin. Front Immunol. (2022) 25 13:884241 doi: 10.3389 10.3389/fimmu.2022.884241 PMC9196036 35711465

[14] Schmitt T Hudemann C Moztarzadeh S Hertl M Tikkanen R Waschke J. Dsg3 epitope-specific signalling in pemphigus. Front Immunol. (2023) 14:1163066. doi: 10.3389/fimmu.2023.1163066 37143675 PMC10151755

[15] Ünlü S Sánchez Navarro BG Cakan E Berchtold D Meleka Hanna R Vural S. Exploring the depths of IgG4: insights into autoimmunity and novel treatments. Front Immunol. (2024) 15:1346671. doi: 10.3389/fimmu.2024.1346671 38698867 PMC11063302

[16] Amendt T Yu P. TLR7 and igM: dangerous partners in autoimmunity. Antibodies (Basel). (2023) 12:4. doi: 10.3390/antib12010004 36648888 PMC9844493

[17] Strandmoe AL Bremer J Diercks GFH Gostyński A Ammatuna E Pas HH. Beyond the skin: B cells in pemphigus vulgaris, tolerance and treatment. Br J Dermatol. (2024) 191:164–76. doi: 10.1093/bjd/ljae107 38504438

[18] Scurtu LG Simionescu O. Soluble factors and receptors involved in skin innate immunity-what do we know so far? Biomedicines. (2021) 9:1795. doi: 10.3390/biomedicines9121795 34944611 PMC8698371

[19] Yamamoto Y Aoyama Y Shu E Tsunoda K Amagai M Kitajima Y. Anti-desmoglein 3 (Dsg3) monoclonal antibodies deplete desmosomes of Dsg3 and differ in their Dsg3-depleting activities related to pathogenicity. J Biol Chem. (2007) 282:17866–76. doi: 10.1074/jbc.M607963200 17428808

[20] Bumiller-Bini-Hoch V Schneider L Pumpe AE Lüders E Hundt JE Boldt ABW. Marked to die-cell death mechanisms for keratinocyte acantholysis in pemphigus diseases. Life (Basel). (2022) 12:329. doi: 10.3390/life12030329 35330080 PMC8948972

[21] Abrikosova VA Mokrushina YA Ovchinnikova LA Larina EN Terekhov SS Baranova MN. Smirnov IV. B cell profiling in patients with pemphigus vulgaris. Acta Naturae. (2023) 15:13–8. doi: 10.32607/actanaturae.11890 PMC10154782 37153513

[22] Papara C Danescu S Rogojan L Leucuta DC Candrea E Zillikens D. Lymphocyte-predominant lesional inflammatory infiltrates of the skin are associated with mucosal-dominant phenotype in pemphigus. J Cutan Pathol. (2023) 50:754–62. doi: 10.1111/cup.14395 36680509

[23] Fang H Li Q Wang G. The role of T cells in pemphigus vulgaris and bullous pemphigoid. Autoimmun Rev. (2020) 19:102661. doi: 10.1016/j.autrev.2020.102661 32942041

[24] Veldman C Pahl A Beissert S Hansen W Buer J Dieckmann D. Inhibition of the transcription factor Foxp3 converts desmoglein 3-specific type 1 regulatory T cells into Th2-like cells. J Immunol. (2006) 176:3215–22. doi: 10.4049/jimmunol.176.5.3215 16493082

[25] Yuan H Zhou S Liu Z Cong W Fei X Zeng W. Pivotal role of lesional and perilesional T/B lymphocytes in pemphigus pathogenesis. J Invest Dermatol. (2017) 137:2362–70. doi: 10.1016/j.jid.2017.05.032 28647348

[26] Ansari MA Singh PK Dar SA Rai G Akhter N Pandhi D. Deregulated phenotype of autoreactive Th17 and Treg clone cells in pemphigus vulgaris after *in-vitro* treatment with desmoglein antigen (Dsg-3). Immunobiology. (2023) 228:152340. doi: 10.1016/j.imbio.2023.152340 36689824

[27] Abulikemu K Hu F Liang J Kang X. Targeting therapy in pemphigus: Where are we now and where are we going? Heliyon. (2023) 9:e16679. doi: 10.1016/j.heliyon.2023.e16679 37292301 PMC10245244

[28] Aslanova M Yarrarapu SNS Syed HA Zito PM. IgA pemphigus. 2024 may 1. In: StatPearls. StatPearls Publishing, Treasure Island (FL (2024). 30085605

[29] Amber KT Valdebran M Kridin K Grando SA. The role of eosinophils in bullous pemphigoid: A developing model of eosinophil pathogenicity in mucocutaneous disease. Front Med (Lausanne). (2018) 5:201. doi: 10.3389/fmed.2018.00201 30042946 PMC6048777

[30] Cipolla GA Park JK Lavker RM Petzl-Erler ML. Crosstalk between signaling pathways in pemphigus: A role for endoplasmic reticulum stress in p38 mitogen-activated protein kinase activation? Front Immunol. (2017) 8:81022. doi: 10.3389/fimmu.2017.01022 PMC5591886 28928733

[31] Petruzzi M Della Vella F Squicciarini N Lilli D Campus G Piazzolla G. Diagnostic delay in autoimmune oral diseases. Oral Dis. (2023) 29:2614–23. doi: 10.1111/odi.14480 36565434

[32] Daltaban Ö Özçentik A Akman Karakaş A Üstün K Hatipoğlu M Uzun S. Clinical presentation and diagnostic delay in pemphigus vulgaris: A prospective study from Turkey. J Oral Pathol Med. (2020) 49:681–6. doi: 10.1111/jop.13052 32516514

[33] Malik AM Tupchong S Huang S Are A Hsu S Motaparthi K. An updated review of pemphigus diseases. Medicina (Kaunas). (2021) 57:1080. doi: 10.3390/medicina57101080 34684117 PMC8540565

[34] Mahmood MN. Direct immunofluorescence of skin and oral mucosa: guidelines for selecting the optimum biopsy site. Dermatopathology. (2024) 11:52–61. doi: 10.3390/dermatopathology11010006 38390848 PMC10885087

[35] Premalatha S Jayakumar S Yesudian P Thambiah AS. Cerebriform tongue-a clinical sign in pemphigus vegetans. Br J Dermatol. (1981) 104:587–91. doi: 10.1111/j.1365-2133.1981.tb08177.x 7236520

[36] Rebello MS Ramesh BM Sukumar D Alapatt GF. Cerebriform cutaneous lesions in pemphigus vegetans. Indian J Dermatol. (2016) 61:206–8. doi: 10.4103/0019-5154.177760 PMC4817450 27057025

[37] Kim JH Kim SC. Paraneoplastic pemphigus: paraneoplastic autoimmune disease of the skin and mucosa. Front Immunol. (2019) 10:1259. doi: 10.3389/fimmu.2019.01259 31214197 PMC6558011

[38] Lepe K Yarrarapu SNS Zito PM. Pemphigus foliaceus. 2023 Aug 8. In: StatPearls. StatPearls Publishing, Treasure Island (FL (2024). 29763004

[39] Rehman A Huang Y Wan H. Evolving mechanisms in the pathophysiology of pemphigus vulgaris: A review emphasizing the role of desmoglein 3 in regulating p53 and the yes-associated protein. Life (Basel). (2021) 11:621. doi: 10.3390/life11070621 34206820 PMC8303937

[40] Valentino A Leuci S Galderisi U Spagnuolo G Mignogna MD Peluso G. Plasma Exosomal microRNA Profile Reveals miRNA 148a-3p Downregulation in the Mucosal-Dominant Variant of Pemphigus Vulgaris. Int J Mol Sci. (2023) 24:11493. doi: 10.3390/ijms241411493 37511259 PMC10380621

[41] Kappius RH Ufkes NA Thiers BH. Paraneoplastic pemphigus. 2023 may 8. In: StatPearls. StatPearls Publishing, Treasure Island (FL (2024). 31536300

[42] de Almeida HL Jr Neugebauer MG Guarenti IM Aoki V. Pemphigus vegetans associated with verrucous lesions: expanding a phenotype. Clinics (Sao Paulo). (2006) 61:279–82. doi: 10.1590/s1807-59322006000300016 16832564

[43] Son YM Kang HK Yun JH Roh JY Lee JR. The neumann type of pemphigus vegetans treated with combination of dapsone and steroid. Ann Dermatol. (2011) 23:S310–3. doi: 10.5021/ad.2011.23.S3.S310 PMC3276784 22346265

[44] Schmidt E Groves R. Immunobullous diseases. In: Griffith C Barker J Bleiker T Hussain W Simpson R;, editors. Rook’s textbook of dermatology, 10 th edition, vol. 2 . Wiley Blackwell, Glasgow, Great Britain (2024). p. 50.1–6.

[45] Paolino G Didona D Magliulo G Iannella G Didona B Mercuri SR. Paraneoplastic pemphigus: insight into the autoimmune pathogenesis, clinical features and therapy. Int J Mol Sci. (2017) 18:2532. doi: 10.3390/ijms18122532 29186863 PMC5751135

[46] Düker I Schaller J Rose C Zillikens D Hashimoto T Kunze J. Subcorneal pustular dermatosis-type IgA pemphigus with autoantibodies to desmocollins 1, 2, and 3. Arch Dermatol. (2009) 145:1159–62. doi: 10.1001/archdermatol.2009.224 19841404

[47] Zeidler C Pereira MP Huet F Misery L Steinbrink K Ständer S. Pruritus in autoimmune and inflammatory dermatoses. Front Immunol. (2019) 10:1303. doi: 10.3389/fimmu.2019.01303 31293565 PMC6598632

[48] Rolader R Daugherty LN Liu Y Feldman RJ. Prevalence and predictors of pruritus in pemphigus compared with bullous pemphigoid: A cross-sectional study. J Am Acad Dermatol. (2020) 83:251–4. doi: 10.1016/j.jaad.2020.01.025 31962093

[49] Okuno S Hashimoto T Yamazaki Y Okuzawa M Satoh T. IL-31 and IL-31 receptor alpha in pemphigus: Contributors to more than just itch? J Dermatol. (2023) 50:927–30. doi: 10.1111/1346-8138.16730 36651089

[50] Karray M Badri T. Pemphigus herpetiformis. 2023 jul 17. In: StatPearls. StatPearls Publishing, Treasure Island (FL (2024). 29494022

[51] Costa LMC Cappel MA Keeling JH. Clinical, pathologic, and immunologic features of pemphigus herpetiformis: a literature review and proposed diagnostic criteria. Int J Dermatol. (2019) 58:997–1007. doi: 10.1111/ijd.14395 30900757

[52] Fuentes-Finkelstein P Barnadas M Gelpi C Puig L. Pemphigus herpetiformis with progression to pemphigus foliaceus: a case report. Actas Dermosifiliogr. (2014) 105:526–8. doi: 10.1016/j.ad.2013.08.009 24168913

[53] Kucukoglu R Atci T Sun GP. Is transition between subtypes of pemphigus possible? A series of pemphigus vulgaris patients showing the transition to pemphigus foliaceus. Bras Dermatol. (2023) 98:787–92. doi: 10.1016/j.abd.2022.09.012 PMC10589459 37357115

[54] Park SG Chang JY Cho YH Kim SC Lee MG. Transition from pemphigus foliaceus to pemphigus vulgaris: case report with literature review. Yonsei Med J. (2006) 47:278–81. doi: 10.3349/ymj.2006.47.2.278 PMC2687642 16642562

[55] Odonwodo A Vashisht P. Bullous systemic lupus erythematosus. 2023 may 22. In: StatPearls. StatPearls Publishing, Treasure Island (FL (2024). 32491377

[56] Chavan SA Sharma YK Deo K Buch AC. A case of Senear-Usher syndrome. Indian J Dermatol. (2013) 58:329. doi: 10.4103/0019-5154.114009 PMC3726916 23919039

[57] Pérez-Pérez ME Avalos-Díaz E Herrera-Esparza R. Autoantibodies in senear-usher syndrome: cross-reactivity or multiple autoimmunity? Autoimmune Dis. (2012) 2012:296214. doi: 10.1155/2012/296214 23320149 PMC3539423

[58] van Beek N Holtsche MM Atefi I Olbrich H Schmitz MJ Pruessmann J. State-of-the-art diagnosis of autoimmune blistering diseases. Front Immunol. (2024) 15:1363032. doi: 10.3389/fimmu.2024.1363032 38903493 PMC11187241

[59] Wallach D. Vintage descriptions of IgA pemphigus. J Eur Acad Dermatol Venereol. (2022) 36:e1012. doi: 10.1111/jdv.18419 35841291

[60] Horvath B Jonkman MF. IgA pemphigus. Autoimmune Bullousd Diseases. (2022) 790:93–98. doi: 10.1007/978-3-030-91557-5_11

[61] Wu H Allan AE Harrist T. Noninfectious vesiculobullous and vesiculopustular diseases in Lever’s histopathology of the skin. Elder DD, editor. Philadelphia: Wolter Kluwer Health (2015). p. 295.

[62] Hashimoto T Komai A Futei Y Nishikawa T Amagai M. Detection of IgA autoantibodies to desmogleins by an enzyme-linked immunosorbent assay: the presence of new minor subtypes of IgA pemphigus. Arch Dermatol. (2001) 137:735–8. doi: 10-1001/pubs.ArchDermatol 11405762

[63] Miyagawa S Hashimoto T Ohno H Nakagawa A Watanabe K Nishikawa T. Atypical pemphigus associated with monoclonal IgA gammopathy. J Am Acad Dermatol. (1995) 32:352–7. doi: 10.1016/0190-9622(95)90402-6 7829739

[64] Min MS Damstetter E Chen AYY. Autoimmune blistering disorders in the setting of human immunodeficiency virus infection. Int J Womens Dermatol. (2018) 4:159–65. doi: 10.1016/j.ijwd.2018.02.002 PMC6116819 30175218

[65] Seo JW Park J Lee J Kim MY Choi HJ Jeong HJ. A case of pemphigus vulgaris associated with ulcerative colitis. Intest Res. (2018) 16:147–50. doi: 10.5217/ir.2018.16.1.147 PMC5797262 29422810

[66] Knabel M Dahiya M Eilers D. 32677 IgA pemphigus in a patient with rheumatoid arthritis and cardiac amyloid successfully treated with oral dapsone. JAAD. (2022) 87:3.815. doi: 10.1016/j.jaad.2022.06.726

[67] Bruijn TVM Geraedts A Vlahu CA Jaspars LH Elshot YS. IgA pemphigus as an immune checkpoint inhibitor-associated skin manifestation. JAAD Case Rep. (2024) 47:41–3. doi: 10.1016/j.jdcr.2024.02.025 PMC11021218 38633887

[68] Sluzevich JC Mutasim D. In xPharm: The Comprehensive Pharmacology Reference. Sci Direct. (2007) 1–6.

[69] Toosi S Collins JW Lohse CM Wolz MM Wieland CN Camilleri MJ. Clinicopathologic features of IgG/IgA pemphigus in comparison with classic (IgG) and IgA pemphigus. Int J Dermatol. (2016) 55:e184–90. doi: 10.1111/ijd.13025 26566588

[70] Cheng HF Tsoi WK Ng MMT Ip WK Ho KM. IgG/IgA pemphigus with differing regional presentations. JAAD Case Rep. (2022) 28:119–22. doi: 10.1016/j.jdcr.2022.03.026 PMC9486352 36147207

[71] Aimo C Corrà A Mariotti E Verdelli A Del Bianco E Bianchi B. IgA pemphigus and Sneddon Wilkinson disease: a spectrum of diseases? Ital J Dermatol Venereol. (2022) 157:456–7. doi: 10.23736/S2784-8671.22.07217-6 36213973

[72] Manjaly P Sanchez K Gregoire S Ly S Kamal K Mostaghimi A. Superficial and bullous neutrophilic dermatoses: sneddon-wilkin, igA pemphigus, and bullous lupus. Dermatol Clin. (2024) 42:307–15. doi: 10.1016/j.det.2023.08.010 38423689

[73] Kerroum S Ammar N Znati K Ismaili N Meziane M Benzekri L. Maladie de Sneddon-Wilkinson: à propos d’un cas [Sneddon-Wilkinson disease: a case report. Pan Afr Med J. (2022) 43:115. doi: 10.11604/pamj.2022.43.115.33116 36721471 PMC9860085

[74] Pile HD Yarrarapu SNS Crane JS. Drug induced pemphigus. 2023 aug 7. In: StatPearls. StatPearls Publishing, Treasure Island (FL (2024). 29763039

[75] Ghaedi F Etesami I Aryanian Z Kalantari Y Goodarzi A Teymourpour A. Drug-induced pemphigus: A systematic review of 170 patients. Int Immunopharmacol. (2021) 92:107299. doi: 10.1016/j.intimp.2020.107299 33418246

[76] Moro F Sinagra JLM Salemme A Fania L Mariotti F Pira A. Pemphigus: trigger and predisposing factors. Front Med (Lausanne). (2023) 10:1326359. doi: 10.3389/fmed.2023.1326359 38213911 PMC10783816

[77] Asdourian MS Shah N Jacoby TV Reynolds KL Chen ST. Association of bullous pemphigoid with immune checkpoint inhibitor therapy in patients with cancer: A systematic review. JAMA Dermatol. (2022) 158:933–41. doi: 10.1001/jamadermatol.2022.1624 35612829

[78] Gholizadeh N Taghavi Zenouz A Eslami H. Pemphigus vulgaris associated with rheumatoid arthritis in a patient not taking penicillamine. J Dent Res Dent Clin Dent Prospects. (2012) 6:33–5. doi: 10.5681/joddd.2012.008 PMC3442446 22991633

[79] De D Shah S Mahajan R Handa S. Pemphigus and pregnancy. Indian Dermatol Online J. (2024) 15:749–57. doi: 10.4103/idoj.idoj_632_23 PMC11444454 39359288

[80] Vičić M Marinović B. Autoimmune bullous diseases in pregnancy: an overview of pathogenesis, clinical presentations, diagnostics and available therapies. Ital J Dermatol Venerol. (2023) 158:99–109. doi: 10.23736/S2784-8671.23.07553-9 37153944

[81] Lan Y Zhang H Jin H. Pregnancy in pemphigus vulgaris: A systematic review. Am J Reprod Immunol. (2024) 91:e13813. doi: 10.1111/aji.13813 38282607

[82] Aguilante C Durán JA Ahumada E Sandoval A. Recién nacido con pénfigo sifilítico en tiempos de pandemia [Newborn with syphilitic pemphigus in pandemic’s time. Rev Chil Infectol. (2021) 38:800–4. doi: 10.4067/s0716-10182021000600800 35506855

[83] Anhalt GJ Kim SC Stanley JR Korman NJ Jabs DA Kory M. Paraneoplastic pemphigus. An autoimmune mucocutaneous disease associated with neoplasia. N Engl J Med. (1990) 323:1729–35. doi: 10.1056/NEJM199012203232503 2247105

[84] Prado R Brice SL Fukuda S Hashimoto T Fujita M. Paraneoplastic pemphigus herpetiformis with IgG antibodies to desmoglein 3 and without mucosal lesions. Arch Dermatol. (2011) 147:67–71. doi: 10.1001/archdermatol.2010.362 21242397

[85] Luo Y Fei X Wang M Yang H Zhang Y Chen Y. Epidemiology of Malignant tumors in patients with pemphigus: an analysis of trends from 1955 to 2021. Clin Exp Med. (2024) 24:100. doi: 10.1007/s10238-024-01354-8 38758217 PMC11101525

[86] Bansari A Wallace JA Yang L Kapoor A. Paraneoplastic pemphigus presenting as a prodrome to aggressive T cell lymphoma. BMJ Case Rep. (2024) 17:e258580. doi: 10.1136/bcr-2023-258580 38839409

[87] Cao L Wang F Du XY Zhu HY Wang L Xu W. Chronic lymphocytic leukemia-associated paraneoplastic pemphigus: potential cause and therapeutic strategies. Sci Rep. (2020) 10:16357. doi: 10.1038/s41598-020-73131-y 33004832 PMC7529904

[88] Grigore M Costache M Simionescu O. Paraneoplastic pemphigus mimicking pemphigus vulgaris associated with castleman disease. Cureus. (2023) 15:e36114. doi: 10.7759/cureus.36114 37065416 PMC10098500

[89] Shirai T Kiniwa Y Ishii N Hashimoto T Senoo Y Urushihata K. Paraneoplastic pemphigus associated with Waldenström’s macroglobulinemia. J Dermatol. (2020) 47:e200–1. doi: 10.1111/1346-8138.15289 32103535

[90] Maldonado F Pittelkow MR Ryu JH. Constrictive bronchiolitis associated with paraneoplastic autoimmune multi-organ syndrome. Respirology. (2009) 14:129–33. doi: 10.1111/j.1440-1843.2008.01397.x 19144057

[91] Antiga E Bech R Maglie R Genovese G Borradori L Bockle B. S2k guidelines on the management of paraneoplastic pemphigus/paraneoplastic autoimmune multiorgan syndrome initiated by the European Academy of Dermatology and Venereology (EADV). J Eur Acad Dermatol Venereol. (2023) 37:1118–34. doi: 10.1111/jdv.18931 PMC10806824 36965110

[92] Svoboda SA Huang S Liu X Hsu S Motaparthi K. Paraneoplastic pemphigus: Revised diagnostic criteria based on literature analysis. J Cutan Pathol. (2021) 48:1133–8. doi: 10.1111/cup.14004 33719070

[93] Tsuji Y Kawashima T Yokota K Tateish Y Tomita Y Matsumura T. Clinical and serological transition from pemphigus vulgaris to pemphigus foliaceus demonstrated by desmoglein ELISA system. Arch Dermatol. (2002) 138:95–6. doi: 10.1001/archderm.138.1.95 11790172

[94] Lévy-Sitbon C Reguiaï Z Durlach A Goeldel AL Grange F Bernard P. Transition phénotypique d’un pemphigus vulgaire en pemphigus superficiel [Transition from pemphigus vulgaris to pemphigus foliaceus: a case report. Ann Dermatol Venereol. (2013) 140:788–92. doi: 10.1016/j.annder.2013.07.013 24315225

[95] España A Koga H Suárez-Fernández R Ohata C Ishii N Irarrazaval I. Antibodies to the amino-terminal domain of desmoglein 1 are retained during transition from pemphigus vulgaris to pemphigus foliaceus. Eur J Dermatol. (2014) 24:174–9. doi: 10.1684/ejd.2014.2277 24776707

[96] Ben Lagha I Ashack K Khachemoune A. Hailey-hailey disease: an update review with a focus on treatment data. Am J Clin Dermatol. (2020) 21:49–68. doi: 10.1007/s40257-019-00477-z 31595434

[97] Vodo D Sarig O Sprecher E. The genetics of pemphigus vulgaris. Front Med (Lausanne). (2018) 5:226. doi: 10.3389/fmed.2018.00226 30155467 PMC6102399

[98] Petzl-Erler ML. Beyond the HLA polymorphism: A complex pattern of genetic susceptibility to pemphigus. Genet Mol Biol. (2020) 43:e20190369. doi: 10.1590/1678-4685-gmb-2019-0369 32639508 PMC7341728

[99] Assaf S Malki L Mayer T Mohamad J Peled A Pavlovsky M. ST18 affects cell-cell adhesion in pemphigus vulgaris in a tumour necrosis factor-α-dependent fashion. Br J Dermatol. (2021) 184:1153–60. doi: 10.1111/bjd.19679 33205400

[100] Vodo D Sarig O Geller S Ben-Asher E Olender T Bochner R. Identification of a functional risk variant for pemphigus vulgaris in the ST18 gene. PloS Genet. (2016) 12:e1006008. doi: 10.1371/journal.pgen.1006008 27148741 PMC4858139

[101] Shetty VM Subramaniam K Rao R. Utility of immunofluorescence in dermatology. Indian Dermatol Online J. (2017) 8:1–8. doi: 10.4103/2229-5178.198774 28217464 PMC5297263

[102] Brar A Sharma A Nauhria S Nauhria S Bhattacharjee A Peela J. Utility of direct immunofluorescence in cutaneous autoimmune bullous disorders. Cureus. (2021) 13:e14562. doi: 10.7759/cureus.14562 34026378 PMC8133519

[103] Mysorekar VV Sumathy TK Shyam Prasad AL. Role of direct immunofluorescence in dermatological disorders. Indian Dermatol Online J. (2015) 6:172–80. doi: 10.4103/2229-5178.156386 PMC4439745 26009711

[104] Dhanabalan RT Ramalingam S Ibrahim SS Ganesan BM Balan LK Thandavarayan P. The utility of immunofluorescence in diagnosing dermatological lesions and its correlation with clinical and histopathological diagnosis in a tertiary health care setup. Indian J Dermatopathology Diagn Dermatol. (2016) 3:63–70. doi: 10.4103/2349-6029.195225

[105] Tsuruta D Ishii N Hamada T Ohyama B Fukuda S Koga H. IgA pemphigus. Clin Dermatol. (2011) 29:437–42. doi: 10.1016/j.clindermatol.2011.01.014 21679872

[106] Porro AM Caetano Lde V Maehara Lde S Enokihara MM. Non-classical forms of pemphigus: pemphigus herpetiformis, IgA pemphigus, paraneoplastic pemphigus and IgG/IgA pemphigus. Bras Dermatol. (2014) 89:96–106. doi: 10.1590/abd1806-4841.20142459 PMC3938360 24626654

[107] Basu K Chatterjee M De A Sengupta M Datta C. Mitra P. A clinicopathological and immunofluorescence study of intraepidermal immunobullous diseases. Indian J Dermatol. (2019) 64:101–5. doi: 10.4103/ijd.IJD_515_17 PMC6440185 30983604

[108] Buch AC Kumar H Panicker N Misal S Sharma Y. Gore CR. A cross-sectional study of direct immunofluorescence in the diagnosis of immunobullous dermatoses. Indian J Dermatol. (2014) 59:364–8. doi: 10.4103/0019-5154.135488 PMC4103273 25071256

[109] Rana D Khurana N Mandal S Sahoo BL. Direct immunofluorescence (DIF) versus immunohistochemical (IHC) staining of complements and immunoglobulins (Ig) in pemphigus group. Indian J Pathol Microbiol. (2024) 167:336–9. doi: 10.4103/ijpm.ijpm_113_23 38427745

[110] Villani AP Chouvet B Kanitakis J. Application of C4d immunohistochemistry on routinely processed tissue sections for the diagnosis of autoimmune bullous dermatoses. Am J Dermatopathol. (2016) 38:186–8. doi: 10.1097/DAD.0000000000000333 25793311

[111] Saschenbrecker S Karl I Komorowski L Probst C Dähnrich C Fechner K. Serological diagnosis of autoimmune bullous skin diseases. Front Immunol. (2019) 10:1974. doi: 10.3389/fimmu.2019.01974 31552014 PMC6736620

[112] Marinović B Fabris Z Lipozencić J Stulhofer Buzina D Lakos Jukić I. Comparison of diagnostic value of indirect immunofluorescence assay and desmoglein ELISA in the diagnosis of pemphigus. Acta Dermatovenerol Croat. (2010) 18:79–83. 20624356

[113] Kridin K Bergman R. The usefulness of indirect immunofluorescence in pemphigus and the natural history of patients with initial false-positive results: A retrospective cohort study. Front Med (Lausanne). (2018) 5:266. doi: 10.3389/fmed.2018.00266 30386780 PMC6199371

[114] Solimani F Didona D Hertl M. Hautfragilität bei blasenbildenden Autoimmundermatosen [Skin fragility in autoimmune blistering diseases of the skin. Dermatologie (Heidelb). (2024) 75(12):924–33. doi: 10.1007/s00105-024-05428-2 39542883

[115] Ramos W Díaz J Gutierrez EL Lazarte JS Bohnett MC Ronceros G. Antidesmoglein 1 and 3 antibodies in healthy subjects of a population in the Peruvian high amazon. Int J Dermatol. (2018) 57:344–8. doi: 10.1111/ijd.13824 29130480

[116] Rai R Ponmariappan L. Anti-desmoglein autoantibody in a patient with bullous pemphigoid - A case report. Indian Dermatol Online J. (2022) 13:794–5. doi: 10.4103/idoj.idoj_45_22 PMC9650757 36386739

[117] Ward KE Steadman L Karim AR Reynolds GM Pugh M Chua W. SARS-CoV-2 infection is associated with anti-desmoglein 2 autoantibody detection. Clin Exp Immunol. (2023) 213:243–51. doi: 10.1093/cei/uxad046 PMC10651225 37095599

[118] Gui H Young PA So JY Pol-Rodriguez M Rieger KE Lewis MA. New-onset pemphigus vegetans and pemphigus foliaceus after SARS-CoV-2 vaccination: A report of 2 cases. JAAD Case Rep. (2022), 27:94–98. doi: 10.1016/j.jdcr.2022.07.002 PMC9270232 35845348

